# Molecular characteristics and improved survival prediction in a cohort of 2023 ependymomas

**DOI:** 10.1007/s00401-023-02674-x

**Published:** 2024-01-24

**Authors:** Lara C. Pohl, Maximilian Leitheiser, Denise Obrecht, Leonille Schweizer, Annika K. Wefers, Alicia Eckhardt, Mark Raffeld, Dominik Sturm, Kristian W. Pajtler, Stefan Rutkowski, Kohei Fukuoka, Koichi Ichimura, Michael Bockmayr, Ulrich Schüller

**Affiliations:** 1 Department of Pediatric Hematology and Oncology, University Medical Center Hamburg-Eppendorf, Hamburg, Germany; 2 Research Institute Children’s Cancer Center Hamburg, Hamburg, Germany; 3 Institute of Pathology, Charité—Universitätsmedizin Berlin, Corporate Member of Freie Universität Berlin and Humboldt-Universität zu Berlin, Berlin, Germany; 4 Institute of Pathology, Ludwig Maximilians University Hospital Munich, Munich, Germany; 5 Institute of Neurology (Edinger Institute), University Hospital Frankfurt, Goethe University, Frankfurt am Main, Germany; 6 German Cancer Consortium (DKTK), Partner Site Frankfurt/Mainz, German Cancer Research Center (DKFZ), Heidelberg, Germany; 7 Frankfurt Cancer Institute (FCI), Frankfurt am Main, Germany; 8 Institute of Neuropathology, University Medical Center Hamburg-Eppendorf, Hamburg, Germany; 9 Department of Radiotherapy and Radiation Oncology, Hubertus Wald Tumor Center—University Cancer Center Hamburg, University Medical Center Hamburg-Eppendorf, Hamburg, Germany; 10 Laboratory of Pathology, National Cancer Institute, National Institutes of Health, Bethesda, MD USA; 11 Hopp Children’s Cancer Center Heidelberg (KiTZ), Heidelberg, Germany; 12 Division of Pediatric Glioma Research, German Cancer Research Center (DKFZ) and German Cancer Consortium (DKTK), Heidelberg, Germany; 13 Department of Pediatric Oncology, Hematology and Immunology, Heidelberg University Hospital, Heidelberg, Germany; 14 Division of Pediatric Neurooncology, German Cancer Research Center (DKFZ) and German Cancer Consortium (DKTK), Heidelberg, Germany; 15 KiTZ Clinical Trial Unit, Department of Pediatric Oncology, Hematology, Immunology and Pulmonology, Heidelberg University Hospital, Heidelberg, Germany; 16 Department of Hematology/Oncology, Saitama Children’s Medical Center, Saitama, Japan; 17 Department of Brain Disease Translational Research, Graduate School of Medicine, Juntendo University, 2-1-1 Hongo, Bunkyo-Ku, Tokyo, 113-8421 Japan

**Keywords:** Ependymoma, DNA methylation, Molecular types, Survival, Machine learning

## Abstract

The diagnosis of ependymoma has moved from a purely histopathological review with limited prognostic value to an integrated diagnosis, relying heavily on molecular information. However, as the integrated approach is still novel and some molecular ependymoma subtypes are quite rare, few studies have correlated integrated pathology and clinical outcome, often focusing on small series of single molecular types. We collected data from 2023 ependymomas as classified by DNA methylation profiling, consisting of 1736 previously published and 287 unpublished methylation profiles. Methylation data and clinical information were correlated, and an integrated model was developed to predict progression-free survival. Patients with EPN-PFA, EPN-ZFTA, and EPN-MYCN tumors showed the worst outcome with 10-year overall survival rates of 56%, 62%, and 32%, respectively. EPN-PFA harbored chromosome 1q gains and/or 6q losses as markers for worse survival. In supratentorial EPN-ZFTA, a combined loss of *CDKN2A *and *B* indicated worse survival, whereas a single loss did not. Twelve out of 200 EPN-ZFTA (6%) were located in the posterior fossa, and these tumors relapsed or progressed even earlier than supratentorial tumors with a combined loss of *CDKN2A/B*. Patients with MPE and PF-SE, generally regarded as non-aggressive tumors, only had a 10-year progression-free survival of 59% and 65%, respectively. For the prediction of the 5-year progression-free survival, Kaplan-Meier estimators based on the molecular subtype, a Support Vector Machine based on methylation, and an integrated model based on clinical factors, CNV data, and predicted methylation scores achieved balanced accuracies of 66%, 68%, and 73%, respectively. Excluding samples with low prediction scores resulted in balanced accuracies of over 80%. In sum, our large-scale analysis of ependymomas provides robust information about molecular features and their clinical meaning. Our data are particularly relevant for rare and hardly explored tumor subtypes and seemingly benign variants that display higher recurrence rates than previously believed.

**Supplementary Information:**

The online version contains supplementary material available at 10.1007/s00401-023-02674-x.

## Introduction

Ependymomas are a heterogeneous group of central nervous system tumors occurring intracranially and in the spinal cord. In the pediatric age group, ependymomas account for 4.6% of all central nervous system tumors [28]. In adults, they make up more than 16% of tumors detected in the spinal cord. Data from the 16-year period between 2001 and 2018 attribute 3277 deaths to ependymal tumors in the United States, independent of whether they received treatment [27].

Effective treatment options for ependymoma are currently limited to tumor resection and radiotherapy. Gross-total resection (GTR) has been previously reported to have a significant effect on the progression-free survival (PFS) and overall survival (OS) of patients. Adjuvant radiotherapy is the standard of care for most patients to avoid a recurrence or a progression of an incompletely resected tumor [4]. The use of chemotherapy is controversial, and its efficacy is being tested in different settings and age groups. Recent studies suggest that chemotherapy could provide a benefit in pediatric patients where GTR was not achieved and build a bridge to a second surgery [24, 36]. On the other hand, especially in childhood ependymoma, adverse effects of therapy and possible cognitive impairments resulting from aggressive treatment strategies need to be considered [26]. However, it has remained challenging to stratify patients by their risk of relapse and adapt the care regimen accordingly. Therefore, improved methods or additional adjuncts to predict a patient’s prognosis and treatment response are needed.

The advancement of DNA methylation profiling in recent years has played a major role in this area and promoted several discoveries about ependymomas. Previously, diagnostics mainly relied on histopathologic review, although the prognostic value of WHO histologic grading and morphological patterns of ependymal tumors was limited. The assignment of WHO grades showed high inter-observer variability, and tumors still progressed heterogeneously [9]. Methylation analysis, on the other hand, provides reliable results quickly and is now established in routine diagnostics.

The discovery of nine types of ependymoma defined by methylation profiling furthered the understanding of these tumors, identified distinct genetic aberrations within the subgroups, and provided a strong predictor of the outcome of patients [30, 35]. Since then, several of these molecular types have been further divided into subtypes, and the MYCN-driven spinal ependymomas have been identified as an additional tenth type of ependymoma [3, 7, 12, 29, 41]. This progress is also reflected in the 2021 WHO Classification of Tumors of the Central Nervous System, which now differentiates between multiple molecularly defined types and advises an integrated diagnosis [22]. However, the molecular classification based on DNA methylation has yet to be routinely established in clinical risk stratification.

The original classification defining nine molecular types resulted from analyzing a cohort of 500 ependymomas, and further studies mainly focused on smaller cohorts of a specific type [2, 3, 7, 12, 29, 30, 34, 41]. While the assignment of molecular types results in a more accurate characterization of these tumors, it also creates the issue of rare tumor types with small case numbers. It remains a challenge to gather reliable information about these rare tumor types. So far, large cohorts to validate previous findings regarding prognostic factors in ependymomas are missing. We assembled a cohort of 2023 ependymomas with clinical information across all molecular types to validate previous findings in a larger setting and closely examine the differences between the types. In our study, we also developed machine learning models to predict the prognosis of a patient based on the methylation profile of the tumor. This approach promises a more direct and personalized diagnostic tool in clinical practice.

## Material and methods

### Patient samples

We assembled a cohort of 2023 ependymomas, for which DNA methylation profiling was performed. For these samples, the class with the highest score in the Heidelberg brain tumor classifier v12b6 was a molecular type of ependymoma [5]. For previously published samples, the raw methylation values and clinical data were collected from Gene Expression Omnibus and the corresponding authors (GSE65362, GSE104210, GSE117130, GSE109379, GSE90496, GSE114523, GSE169265, GSE224218, GSE182707, GSE184900, GSE196013, GSE215240) [3, 5, 7, 11, 29, 30, 32, 36, 38–40]. Multiple samples of the same patient were identified via SNP analysis, and only one sample was included in our analyses. Samples that were annotated as relapse or metastasis were excluded from the dataset. An overview of the cohort, including molecular features and clinical covariates, is shown in Supplementary Table 1.

The 287 previously unpublished idat files were obtained from the HIT MED database and collaborating institutions in Hamburg, Heidelberg, Munich, the USA, and Japan (Suppl. Table 1). Clinical samples were collected in the participating institutions after consent was obtained from each patient in accordance with the protocols approved by the local review boards.

### DNA methylation analysis

DNA was isolated using the ReliaPrep™ FFPE gDNA Miniprep System (Promega) according to the manufacturer’s instructions. About 100–500 ng of DNA was used for bisulfite conversion by the EZ DNA Methylation Kit (Zymo Research). The DNA Clean & Concentrator-5 (Zymo Research) and the Infinium HD FFPE DNA Restore Kit (Illumina) were employed to clean and restore the converted DNA. Finally, Infinium BeadChip arrays (Illumina) were used to quantify the methylation status on an iScan (Illumina).

### Data processing

The data were processed using the statistical programming language *R* [33]. The raw idat files for all tumors were imported and preprocessed with the package *minfi,* and noob normalization was performed [1, 10]. Samples with a detection *p*-value > 0.05 were excluded for quality control. Beta values were computed for further analysis. Only CpG sites present on both the 450k and EPIC BeadChips were kept, ensuring equal conditions. CpG sites located on sex chromosomes and associated with SNPs and cross-reactive sites were excluded as described previously [31]. To avoid batch effects between the 450k and EPIC BeadChips, the differentially methylated sites (absolute mean difference > 0.2, two-sided *t*-test *p*-value < 0.01 after Bonferroni correction) were also excluded. The final dataset consisted of *n* = 402,873 CpG sites.

Copy number variation profiles were generated using the *conumee* package [15]. Positions of genes and chromosome arms were identified with the annotation for Illumina’s 450k and EPIC BeadChip arrays [13, 14]. For chromosome arm gains and losses, a threshold of 0.1 was set. For focal gains and losses, a threshold of 0.4 was employed. No distinction was made between heterozygous and homozygous loss/gain of focal and chromosome arm-wise changes, as there are currently no reliable thresholds defined for this in CNV profiles. Immune infiltration scores were computed using the *DIMEimmune* method [37].

### Statistics

Heatmaps were generated with the *ComplexHeatmap* (RRID:SCR_017270) package. Hierarchical clustering on the whole dataset was performed on the 10,000 most variable CpG sites with Euclidean distancing and average linkage. *K*-means clustering was performed for the EPN-PFA subset of the data with the 10,000 most variable CpG sites, *k* = 9, Euclidean distancing, and average linkage. For Uniform Manifold Approximation and Projection (UMAP) analysis of all tumors, the package *umap* (RRID:SCR_018217) was used with 30 nearest neighbors and a minimum distance of 0.5. The *survival* (RRID:SCR_021137) and *survminer* (RRID:SCR_021094) packages were used to generate Kaplan–Meier estimators. The significance was determined using the log-rank test. To generate confusion matrices, the package *cvms* was used.

### Machine learning

The Support Vector Machine (SVM) was set up using the R package *e1071*. The SVM predicted binary 5-year PFS status (no evidence of disease/progression (NED), relapse) from the 10,000 most variable CpG sites in a 10 × 10 nested cross-validation for hyperparameter optimization and estimation of model performance [23]. The class-balanced folds were created using the *createFolds* function from the R package *caret* (RRID:SCR_021138). The choice of dimensionality was validated by a saturation of the log loss decline for inner fold predictions. Dimension reduction was performed in each iteration of the outer loop to avoid data leakage. The optimal hyperparameter set was selected by minimal log loss in an extensive grid search with a linear and RBF kernel, *C* = 2^–5,…,5^, and gamma = 2^–5, …,5^ × 1/√(#CpG sites). The SVM was configured to return probability scores for each class. Predictions were derived from these probability scores by choosing the class with the maximum probability score. For an improved prediction, the samples with a high prediction probability in the SVM were selected, as shown previously [16]. Samples below the threshold of 0.3 or above the threshold of 0.7 for class “relapse” were included.

Further, we developed and evaluated Kaplan–Meier estimators to predict binary 5-year PFS status on the same outer fold structure as used for the SVM. For this, 5-year PFS probability was estimated for each molecular type and subtype on the training sets using the Kaplan–Meier method. The predictions “NED” and “relapse” were then assigned to the molecular (sub-)type by a probability threshold of 0.5. On the respective test sets, each case was then predicted based on its molecular (sub-)type.

In addition to these models, we used a logistic regression model using the *glm* function to predict 5-year PFS in the same cross-validation setting based on clinical risk factors and CNV data. The included factors were age, sex, and extent of resection, as well as the copy number variations chromosome 1q gain, 6q loss, and *CDKN2A/B* loss. We then developed an integrated logistic regression model that combined the clinical risk factors, CNV data, and the probability scores from the SVM based on methylation. The inner fold prediction scores from the SVM model were used to train the logistic regression model, while testing was performed on the prediction scores from the ten outer folds. Thresholding was performed analogously to the method explained above.

### Data availability

The methylation data of the 2023 analyzed ependymomas are deposited at Gene Expression Omnibus under the accession GSE243240.

## Results

### Study dataset

A cohort of 2023 ependymomas was collected retrospectively, including published as well as unpublished methylation data. DNA methylation-based tumor classification (www.molecularneuropathology.org; version v12b6) retrieved the highest score for a methylation class of ependymal tumors for all cases. All ten currently known molecular types were represented in the dataset, with posterior fossa group A ependymoma (EPN-PFA) accounting for almost half of the tumors (*n* = 969). We identified 308 posterior fossa group B tumors (EPN-PFB) and 228 *ZFTA*-fusion positive ependymoma (EPN-ZFTA). Further, 181 tumors were classified as myxopapillary ependymoma (MPE) and 143 as spinal ependymoma (SP-EPN). Fewer tumors were available for the subependymoma class, with 119 assigned to the posterior fossa (PF-SE), 26 in the supratentorial compartment (ST-SE), and 15 in the spinal area (SP-SE). For the group of *MYCN*-amplified spinal (EPN-MYCN) and the *YAP1*-fusion positive (EPN-YAP) ependymoma, 17 tumors were identified for each group (Table 1).

**Table 1:** Overview of included studies and patient data

Source	No. of included cases
Pajtler et al., Cancer Cell (2015)	151
Cavalli et al., Acta Neuropathol (2018)	159
Fukuoka et al., Acta Neuropathol Commun (2018)	77
Pajtler et al., Acta Neuropathol (2018)	500
Capper et al., Nature (2018)	386
Ghasemi et al., Acta Neuropathol (2019)	6
Raffeld et al., Acta Neuropathol Commun (2020)	7
Thomas et al., Acta Neuropathol (2021)	31
Ritzmann et al., Neuro Oncol (2022)	35
Pratt et al., Acta Neuropathol (2022)	7
Bockmayr et al., Neuro Oncol (2022)	127
Träger et al., Neuro Oncol (2023)	118
Sturm et al., Nat Med (2023)	79
HIT MED	248
Others	92
Total	2023
Age [years]	
Range, Median	0–81, 8
< 4	608
4–18	539
> 18	602
Not available	274
Sex	
Female	761
Male	1053
Not available	209
Ratio (male: female)	1.38: 1
Localization	
Supratentorial	246
Posterior fossa	1357
Spinal	326
Not available	94
WHO grade	
1	82
2	489
3	592
4	1
Not available	859
Molecular type	
EPN-ZFTA	228
ZFTA-RELA-A	199
ZFTA-RELA-B	3
ZFTA-FUS-C	17
ZFTA-FUS-D	5
ZFTA-FUS-E	4
EPN-YAP	17
ST-SE	26
EPN-PFA	969
PFA-1a	146
PFA-1b	126
PFA-1c	138
PFA-1d	83
PFA-1e	118
PFA-1f	59
PFA-2a	147
PFA-2b	127
PFA-2c	25
EPN-PFB	308
PFB-1	121
PFB-2	53
PFB-3	70
PFB-4	47
PFB-5	17
PF-SE	119
SP-EPN	143
EPN-MYCN	17
MPE	181
MPE-A	63
MPE-B	108
SP-SE	15
SP-SE-A	12
SP-SE-B	3

The patient’s age at diagnosis ranged from 0 to 81 years, with a median of 8 years. The cohort encompassed 608 infants younger than 4 years of age and 539 children and adolescents between the ages of 4 and 18 years at diagnosis. We also included 602 adult patients, and for 274 patients, there were no data available regarding age (Table 1, Suppl. Table 1). For the full cohort, there was a significant difference in survival between different age groups. With increasing age of the patient, progression-free and OS probability was higher (*p* < 0.0001) (Suppl. Fig. 1). For the molecular types, significant effects of patient age were only found for the PFS and the OS of SP-EPN patients (*p* = 0.001 and *p* = 0.02, Suppl. Fig. 1).

The sex ratio was 1.38:1 with 1053 male and 761 female patients (Table 1). Female patients appeared to have a slightly better prognosis, both regarding the PFS and OS (*p* = 0.004 and *p* = 0.001, Suppl. Fig. 2). When stratifying by molecular type, female sex was a significant factor for improved survival for EPN-PFA tumors (PFS: *p* = 0.0004, OS: *p* = 0.005, Suppl. Fig. 2). In the EPN-YAP group, PFS data were only available for one male patient, who showed an early progression and thus a significantly worse survival compared to the female patients (*p* = 0.008, Suppl. Fig. 2).

Two hundred and forty-six tumors were annotated to be located in the supratentorial compartment and 1357 in the posterior fossa. For 326 tumors, the localization was described as spinal. In the previous histopathological analysis, 82 samples were assigned CNS WHO grade 1. Further, 489 tumors were classified as grade 2 tumors, 592 as grade 3, and one tumor as grade 4. To acknowledge the 2021 WHO Classification of Tumors of the CNS, WHO tumor grades are shown in Arabic numerals, even though the assignment was based on criteria defined by the respective applicable version of the WHO classification at the time of diagnosis [20, 21]. According to the data we collected, 617 patients received a GTR, and in 352 patients, only a STR was achieved (Table 1). The resection status had a significant effect on both the PFS and OS with an improved outcome for patients in whom a GTR was achieved when regarding the whole cohort (log-rank *p* < 0.0001, Suppl. Fig. 3). Regarding the OS of the individual molecular classes, the resection status only had a significant effect in the EPN-PFA subset (*p* < 0.0001, Suppl. Fig. 3). For the PFS, it was significant in EPN-PFA (*p* < 0.0001), MPE (*p* < 0.0001), EPN-PFB (*p* = 0.0003), and PF-SE (*p* = 0.005), but not for the other types (Suppl. Fig. 3).

### DNA methylation analysis

**Fig. 1:**
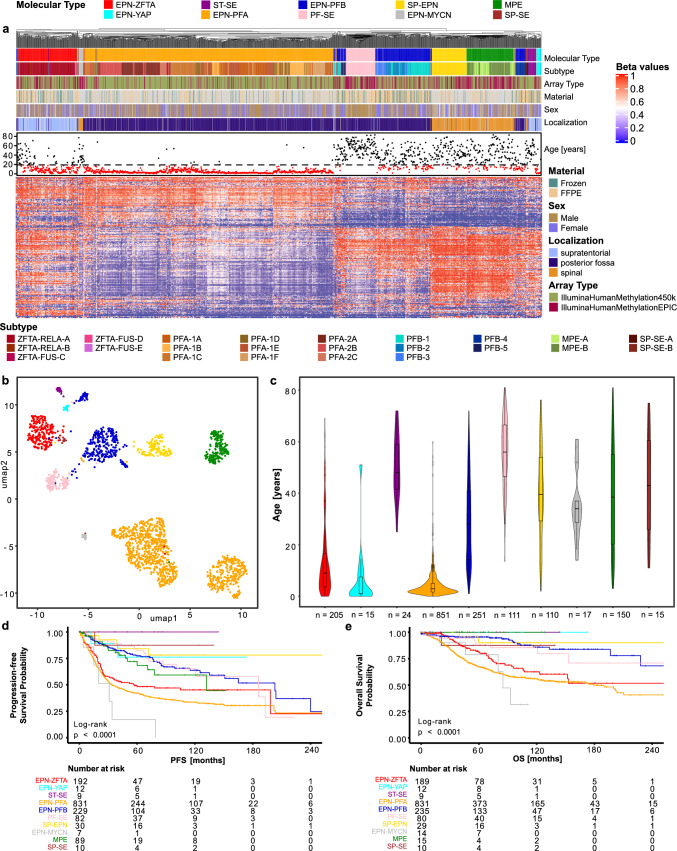
Ten molecular classes of ependymoma defined by DNA methylation-based class prediction form distinct clusters in a large new cohort. **a** Heatmap showing the hierarchical clustering of the DNA methylation profiles of 2023 ependymomas. Each column represents one sample, and the rows show the 10,000 most variable CpG sites. Methylated (red) and unmethylated (blue) sites (beta values) are depicted by a color scale as shown. The associated subtype, array type, material, sex, age, and localization are indicated. **b** Uniform Manifold Approximation and Projection (UMAP) of the ependymoma cohort samples (*n* = 2023) on the 10,000 most variable CpG sites. Individual samples are color-coded according to the respective molecular class as defined by random forest-based class prediction. **c** Violin plot showing the patient age at diagnosis across the ten molecular types. **d** Progression-free survival (PFS) across the ten molecular types. **e** Overall survival (OS) across the ten molecular types

To validate the robustness of the previously defined ten molecular classes by DNA methylation-based tumor classification, we performed a hierarchical clustering analysis of DNA methylation data from all tumors (Fig. 1a). Most of the tumors that were assigned to the same molecular class by automated (random forest-based) class prediction, clustered together except for the EPN-PFB, which split up into three distinct clusters. The three clusters partially fit the previously described five subtypes of EPN-PFB [7]. The subtype PFB-4 formed a cluster separate from the other subtypes and was closest to the MPE and SP-SE subgroups. Of the other two clusters, the larger one comprised samples from the subtypes 1, 2, and 3. The smaller one encompassed tumors from subtypes 1, 3, and 5. The PF-SE showed a close relation to these two clusters while clustering separately from the other subependymomas. The molecular type of EPN-PFA has previously been further subdivided into two subgroups, PFA-1 and PFA-2, as well as nine subtypes, PFA-1a-f and PFA-2a-c [29]. For these subtypes of the EPN-PFA, partial subclustering was found in the analysis of the entire dataset. In this analysis of the whole cohort, the samples showed a distinction between the PFA-1 and PFA-2 groups. However, contrary to previous results, the nine subtypes presented a rather mixed picture and showed no clear separation for most subtypes in this analysis. For the subtypes of the EPN-ZFTA group that are included in version v12b6 of the brain tumor classifier, most tumors were classified to the ZFTA-RELA-A subtype with only a few samples to represent the other subtypes [41]. These showed partial clustering when analyzed with the full cohort (Fig. 1a). No major differences in clustering were found when only including samples with scores above 0.7 or 0.9 for a subtype of ependymoma in the random-forest-based class prediction (Suppl. Fig. 4).

In a UMAP, the molecular types showed distinct groups. Again, the EPN-PFB formed three groups, a bigger one and two smaller ones. The EPN-PFA split into two big groups corresponding to PFA-1 and PFA-2, as previously shown [29] (Fig. 1b).

Analyzing the age distribution of the molecular types showed three types that mainly occurred in the pediatric age group: EPN-ZFTA, EPN-YAP, and EPN-PFA. The molecular types of EPN-PFB, SP-EPN, and MPE encompassed a large age group ranging from pediatric to elderly patients. The subependymomas were mostly limited to the adult age group. The EPN-MYCN group mainly encompassed middle-aged patients. The median for this group was around 35 years of age, which is slightly lower than for other spinal ependymoma subgroups (Fig. 1c).

Survival analyses including all molecular types showed a dismal prognosis for EPN-MYCN, EPN-PFA, and EPN-ZFTA patients. The 10-year OS after the initial diagnosis was only 56% and 62% after a diagnosis of EPN-PFA and EPN-ZFTA, respectively. For the EPN-MYCN group, the 10-year OS rate was only 32%. Relapse or progression was even more frequent, with 50% of the EPN-MYCN tumors already recurring after 31 months. For EPN-PFA and EPN-ZFTA, the 5-year PFS rates were 42% and 48%, respectively. The MPE, SP-EPN, and PF-SE also relapsed quite frequently, with 5-year PFS rates of 68%, 84%, and 82%, respectively. The OS was not impaired. The EPN-PFB had an impaired 5-year progression-free and a reduced 10-year OS of 77% and 86%, respectively (Fig. 1d, e).

The molecular types showed differing immune signatures with CD4 + , CD8 + , and tumor-infiltrating lymphocytes (TIL) in significantly different levels (Kruskal–Wallis test, CD4: *p* = 5.9 × 10^–6^; CD8: *p* < 2.2 × 10^–16^; TIL: *p* < 2.2 × 10^–16^, Suppl. Fig. 5).

### ZFTA ependymoma in the posterior fossa is associated with worse survival

**Fig. 2:**
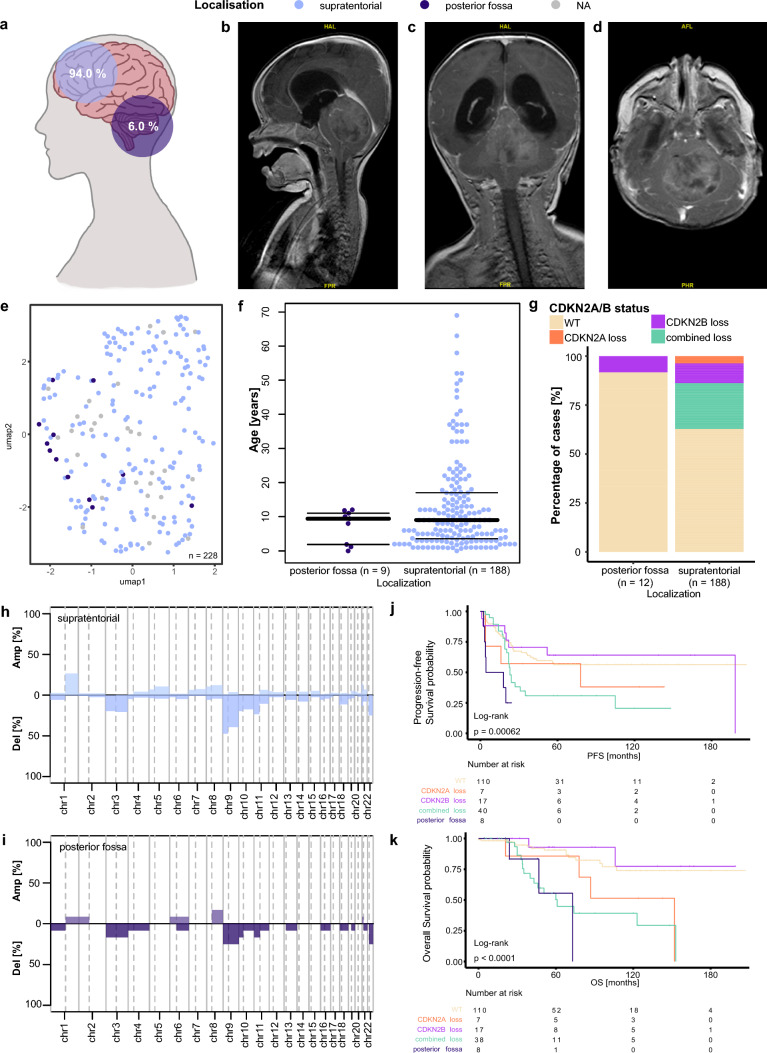
Clinical and molecular characteristics of supratentorial and posterior fossa EPN-ZFTA. **a** Distribution of supratentorial and posterior fossa localization of ZFTA tumors (*n* = 200). **b**–**d** MRI images of a ZFTA tumor located in the posterior fossa: **b** sagittal, **c** coronal, **d** transversal. **e** UMAP of ZFTA tumors (*n* = 228). **f** Age distribution across the localizations in a beeswarm plot. **g** Distribution of *CDKN2A/B* loss across the localizations. **h**–**i** Overview of chromosome arm-wise copy number alterations in supratentorial and posterior fossa tumors. **j** PFS of supratentorial tumors with *CDKN2A/B* alterations and posterior fossa tumors. **k** OS of supratentorial tumors with *CDKN2A/B* alterations and posterior fossa tumors

EPN-ZFTA are generally regarded as supratentorial tumors, although small case numbers of tumors located in the posterior fossa have been previously reported [8, 17]. In our dataset, we identified 12 cases of EPN-ZFTA ependymoma, which were located in the posterior fossa, making up 6% of the EPN-ZFTA tumors in our cohort (Fig. 2a). Radiological imaging of one of these patients is shown in Fig. 2b–d. The 12 samples were mixed with the other samples of the EPN-ZFTA cluster in our clustering analyses of the whole dataset and in a separate analysis of the EPN-ZFTA samples (Fig. 2e). Data on the age at diagnosis were available in nine posterior fossa cases. Three of the infratentorial tumors occurred in infants and the other six in children around the age of 10 years. For the supratentorial tumors, 77% occurred in children and adolescents, whereas 23% were identified in adults (Fisher test: *p* = 0.2, Fig. 2f). Out of the 12 infratentorial EPN-ZFTA tumors, 11 showed a flat CNV profile for *CDKN2A* and *CDKN2B*. Only one sample showed an isolated *CDKN2B* loss. The supratentorial EPN-ZFTA tumors showed a heterogeneous picture when inferring the *CDKN2A/B* status from CNV analyses. Most tumors had a flat profile, but almost a quarter of the tumors harbored a combined loss of *CDKN2A* and *CDKN2B*. There were another 10% of tumors with an isolated *CDKN2B* loss and only seven tumors with a *CDKN2A* loss alone (Fig. 2g). Overall, the copy number profile of the EPN-ZFTA tumors of the posterior fossa showed a mean aberration of 3/44 chromosome arms, while the profile of the supratentorial tumors showed a mean aberration of 5.4/44 chromosome arms. Both showed a high percentage of tumors with a loss of chromosome 9, which is also where *CDKN2A* and *CDKN2B* are located. In supratentorial EPN-ZFTA ependymoma, the tumors with a loss of chromosome 9 made up almost 50%, whereas only 25% of the posterior fossa tumors showed a loss here. In the supratentorial group, about 25% of tumors showed a gain of chromosome 1q, whereas only one of the posterior fossa tumors showed a chromosome arm gain here (Fig. 2h, i).

Survival data were available for eight of the posterior fossa EPN-ZFTA patients and for 174 patients (PFS) and 172 patients (OS) for the supratentorial tumors. The patients with posterior fossa tumors showed a worse PFS and OS when compared to the supratentorial cases, while seven out of the eight tumors had a flat CNV profile for *CDKN2A* and *CDKN2B*. About 75% of the posterior fossa tumors had already relapsed, and only 56% of patients were alive five years after the initial diagnosis. In the supratentorial EPN-ZFTA group, a significant OS difference was present between the tumors with a combined *CDKN2A/B* loss and those with a flat profile (*p* = 0.004). For patients whose tumors harbored a combined loss, this resulted in a 5-year PFS and a 10-year OS of 31% and 39%, respectively. On the other hand, cases that did not show a loss of *CDKN2A* or *CDKN2B* had a 5-year PFS of about 56% and a 10-year OS of 74%. An isolated loss of either *CDKN2A* or *CDKN2B* did not significantly impact the PFS or OS, compared to the tumors without a loss (Fig. 2j, k).

A high or low infiltration with immune cells did not have a significant effect on the survival in the EPN-ZFTA group. There was also no difference between the tumors with high and low mean methylation when splitting either at the median or the best statistical cutoff (Suppl. Fig. 5).

### Combined gain of chromosome 1q and loss of 6q is associated with poor survival in EPN-PFA

**Fig. 3:**
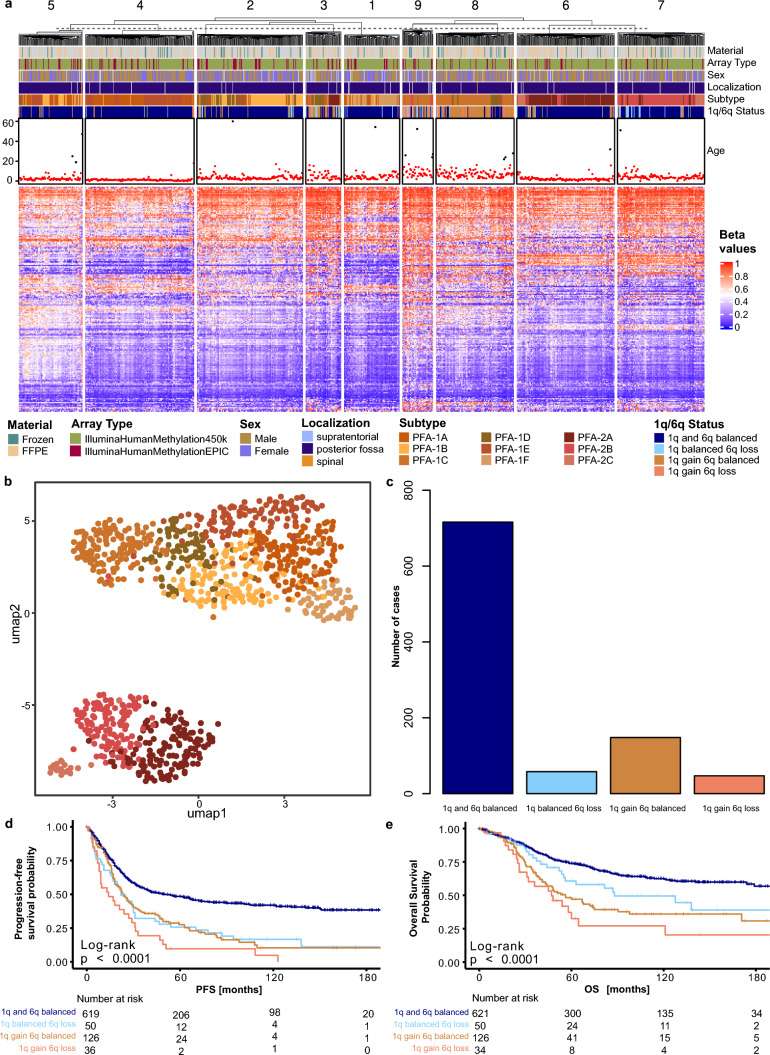
Combined 1q gain and 6q loss leads to worse outcomes in EPN-PFA. **a** Heatmap showing the *k*-means clustering (*k* = 9) of 969 PFA ependymomas. Each column corresponds to one sample, and the rows represent the 10,000 most variable CpG sites. Methylated (red) and unmethylated (blue) sites (beta values) are indicated by a color scale as shown. The associated material, array type, sex, localization, subtype, 1q and 6q status, as well as the patient’s age at diagnosis, are shown. **b** Unsupervised clustering of PFA samples (*n* = 969) in a UMAP using the 10,000 most variable CpG sites. Individual samples are color-coded according to their subtype. **c** Bar plot showing the distribution of chromosome 1q gain and 6q loss status. **d** PFS according to the 1q and 6q status. **e** OS according to the 1q and 6q status

Previous studies revealed two subgroups of posterior fossa group A ependymoma, PFA-1 and PFA-2, and nine distinct subtypes, PFA-1a-f and PFA-2a-c, of EPN-PFA [29]. To validate these previous findings, we conducted a *k*-means analysis with *k* = 9 on all samples from our cohort that were identified as PFA ependymoma and assigned a subtype by the Heidelberg brain tumor classifier. In the resulting heatmap, a distinct separation between the PFA-1 and PFA-2 subgroups is visible. The PFA-2 subgroup is mostly divided into clusters 6 and 7. All clusters show a grouping of different subtypes, and there is no subtype that completely separates itself from the others. Overall, the clustering of the nine EPN-PFA subtypes appears relatively unstable in our analysis. Regarding the chromosome 1q and 6q status, 88% of the tumors of the PFA-1c subtype showed either an isolated 1q gain (56%), an isolated 6q loss (8%), or a combined 1q gain and 6q loss (24%). For the other subtypes, the majority of tumors show a balanced status for the two chromosome arms, with only a few tumors showing aberrations. The age distribution confirms that most patients with an EPN-PFA tumor belong to the pediatric age group. The few adult patients are distributed relatively evenly between the nine clusters. In cluster 4, which mostly encompasses the subtypes 1a and 1e, the age distribution appears to be relatively homogenous with most patients in the lower pediatric age range. Cluster 8 on the other hand shows more variability and many patients in the adolescent age group (Fig. 3a).

A UMAP analysis showed a strong separation between the PFA-1 and PFA-2 subgroups. Inside the PFA-1 group, the PFA-1c and PFA-1f subtypes show the least overlap with the other subtypes, whereas the other subtypes do not distinguish themselves as clearly. In the PFA-2 group, the subtypes PFA-2a and PFA-2b lie close together and show some overlap. The PFA-2c samples form a quite distinct and isolated group from the other PFA-2 tumors (Fig. 3b).

The bar plot in Fig. 3c illustrates the distribution of the chromosome 1q and 6q status across the EPN-PFA tumors. Most samples show a balanced status for both chromosomes 1q and 6q. In 148 tumors, we identified an isolated 1q gain. In another 58 tumors, we found an isolated 6q loss, and 47 tumors showed a combined gain of chromosome 1q and loss of chromosome 6q (Fig. 3c).

Both, a gain of chromosome 1q and a loss of chromosome 6q have previously been shown to be associated with poor OS and PFS in EPN-PFA patients [2]. A survival analysis in our cohort revealed that an isolated 1q gain and an isolated 6q loss have a similar impact on the PFS of patients and lead to worse outcomes than a balanced chromosomal status. The combined loss of chromosome 6q and gain of 1q is associated with an even more impaired PFS. Five-year PFS in patients with a balanced status is 49%, for patients with either 1q gain or 6q loss, it is 28% and 26%, respectively, and for patients with a combined 1q gain and 6q loss, it is 10% (Fig. 3d). Similar to the PFS, the OS is best in patients without chromosomal aberrations on chromosome 1q and 6q, roughly 74% at 5 years after initial diagnosis. For patients with only a 6q loss, the 5-year OS is about 61%, and for patients with a 1q gain, it is 47%. Again, patients with a combined 1q gain and 6q loss showed the worst outcome with a 5-year OS rate of only 33% (Fig. 3e).

The infiltration with immune cells did not influence the OS. The mean methylation did not prove to be a significant factor when separating the group at the median but showed a significant result when splitting at the best statistical cut-off (*p* = 0.003, Suppl. Fig. 5).

### Survival prediction based on most variable CpG sites achieves higher accuracy than prediction based on molecular subtypes

The definition of molecular types and subtypes has allowed clinicians to give more informed estimates of the survival probability and risk of recurrence to the affected patients. We compared multiple different approaches to predict the binary 5-year PFS status (NED, relapse) in this cohort. Kaplan–Meier estimators based only on either molecular type or subtype, as well as logistic regression models based on clinical risk factors and copy number alterations, are used to model the current decision-making in the clinical setting. In addition to that, we developed an SVM based on DNA methylation profiles and created an integrated model that combines clinical factors, copy number alterations, and methylation scores.

**Fig. 4:**
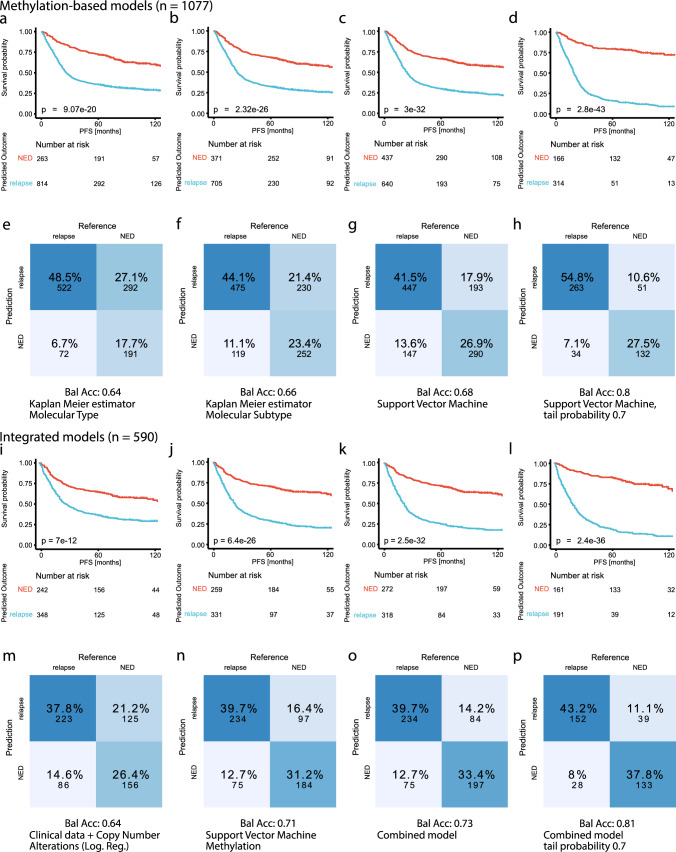
Comparison of molecular subtyping and machine learning models in survival prediction. **a**–**d** PFS stratified by the predicted 5-year PFS based on: **a** the molecular type, **b** the molecular subtype, **c** the Support Vector Machine (SVM), **d** the SVM predictions with a probability score below 0.3 or above 0.7. **e**–**h** Confusion matrices and balanced accuracy for the predicted 5-year PFS based on: **e** the molecular type, **f** the molecular subtype, **g** the SVM, **h** the SVM predictions with a probability score below 0.3 or above 0.7. **i**–**l** PFS stratified by the predicted 5-year PFS from the integrated models based on: **i** clinical data and copy number alterations, **j** the SVM, **k** the combined logistic regression model, **l** the predictions from the combined model with a probability score below 0.3 or above 0.7. **m**–**p** Confusion matrices and balanced accuracy for the predicted 5-year PFS from the integrated models based on: **m** clinical data and copy number alterations, **n** the SVM, **o** the combined logistic regression model, **p** the predictions from the combined model with a probability score below 0.3 or above 0.7. NED, no evidence of disease/progression

The SVM was trained on the 10,000 most variant CpG sites of its training sets, since this number of dimensions proved sufficient for a saturation in log loss score on this dataset (Suppl. Fig. 6) and has been established as a standard value in DNA methylation analysis [18, 23]. Further, the SVM, without additional calibration, showed high reliability when comparing the predicted probability scores to the actual accuracy (Suppl. Fig. 6). Figure 4 shows the result of the application of these methods to the full ependymoma cohort in a (nested) cross-validation setup. The Kaplan–Meier curves model the PFS probability, grouped by the predicted outcome for each method. With the Kaplan–Meier estimator based on molecular type, 814 patients were predicted to relapse within the first 5 years after the initial diagnosis, and 263 patients were assigned as NED. The log-rank test showed a significant difference between the survival curves of the predicted outcomes (*p* = 9.1 × 10^–20^, Fig. 4a). Using molecular subtype instead, the Kaplan–Meier estimator predicted 705 as relapse and 371 cases as NED. The survival curves differed even more for this model as represented by the *p*-value of the log-rank test (*p* = 2.3 × 10^–26^, Fig. 4b). The SVM predicted “relapse” for 640 cases and “NED” for 437 cases. This prediction separated the classes even better and led to a high significance (*p* = 3.0 × 10^–32^, Fig. 4c). Rejecting cases with low prediction probability further improved the results of the SVM. Overall, 314 cases remained for the prediction class “relapse” and 166 for “NED”. This method achieved the highest significance in separating the two prediction classes (*p* = 2.8 × 10^–43^, Fig. 4d).

The Kaplan–Meier estimator based on the molecular type achieved an overall accuracy of 66.2% and a balanced accuracy of 63.7%. The confusion matrix shows a tendency to predict the majority of cases as relapsed after 5 years (Fig. 4e). The same method based on the molecular subtype results in an accuracy of 67.6% and a balanced accuracy of 66.3% (Fig. 4f). The SVM yielded better results and showed less tendency to overfit towards the majority class. It achieved an accuracy of 68.4% and a balanced accuracy of 67.6% (Fig. 4g). We were able to further refine this result when selecting the samples with a high prediction probability and achieved an overall accuracy of 82.3% and a balanced accuracy of 80.3% (Fig. 4h). However, this accuracy was only possible on about 45% of the data, which achieved a high enough probability score (Suppl. Fig. 6). UMAP plots mapping the true and false SVM predictions, and the assigned prediction probability scores can be found in Supplementary Fig. 6.

We achieved similar results for the prediction of the 10-year OS in this cohort with slightly reduced accuracy scores overall. Here, the SVM was also able to separate the samples better than the Kaplan–Meier estimators based on the molecular types and subtypes. For this prediction, fewer data were available, making the prediction more challenging (Suppl. Fig. 7).

For the logistic regression model, we used the following clinical risk factors and copy number alterations: age groups, sex, extent of resection, 1q gain, 6q loss, *CDKN2A* loss, and *CDKN2B* loss. These are known clinical risk factors, and our previous univariate analyses already supported their relevance to the PFS of ependymoma patients (Suppl. Figs. 1, 2, 3). The copy number alterations also correspond to PFS changes in certain ependymoma molecular types (Figs. 2, 3). For these models, 590 fully annotated cases with all relevant factors were available. The Kaplan–Meier curves based on the predictions of the logistic regression using clinical data and copy number alterations differed significantly, showing that 5-year PFS prediction is possible even on relatively limited data (*p* = 7 × 10^–12^, Fig. 4i). The results from the SVM based on methylation yielded an even better log-rank score when comparing the Kaplan–Meier curves (*p* = 6.4 × 10^–26^, Fig. 4j). The combination of clinical factors, CNV, and methylation in one logistic regression further improved the prediction results (*p* = 2.5 × 10^–32^, Fig. 4k). As with the SVM, thresholding of the probability scores led to an improvement in the separation of the Kaplan–Meier curves and is reflected in a low *p*-value in the log-rank test (*p* = 2.4 × 10^–36^, Fig. 4l).

The model based on clinical and CNV data achieved an overall accuracy of 64.2% and a balanced accuracy of 63.8%, similar to the accuracies achieved with the prediction based on molecular types (Fig. 4m). The subset of cases had an accuracy of 70.8% and a balanced accuracy of 70.6% in the prediction based on methylation alone. On average, the SVM performed slightly better on these cases than on all cases where data on the 5-year PFS was available (Fig. 4n). The integrated model resulted in a further improvement with an overall accuracy of 73.1% and a balanced accuracy of 72.9% (Fig. 4o). We were able to achieve higher accuracies when excluding cases with low probability scores, resulting in an accuracy and balanced accuracy of 81% and 80.9%, respectively (Fig. 4p).

## Discussion

Our study emphasizes the achievements made in recent years in the research surrounding ependymomas. Furthermore, several novel aspects are described. First, we provide a very large clinically well-annotated series of ependymomas that allows further insights into frequencies, stability, commonalities, and discrepancies between types and subtypes. Our series outnumbers previous cohorts by far, and all molecular data are freely available to the public. Furthermore, we demonstrate that ZFTA ependymomas may occur in the posterior fossa, a finding that is of diagnostic and clinical importance and goes beyond the description of single case reports. We also report novel data concerning the clinical meaning of *CDKN2A/B* loss in ZFTA ependymomas. Further, we showed that SVMs are a reliable adjunct tool for predicting prognosis in ependymoma patients.

The previously identified ten molecular types showed a high grade of robustness in our large cohort of 2023 samples, with the exception of EPN-PFB, further highlighting the heterogeneity of this group [7, 12, 30]. In contrast to the molecular types, the previously defined subtypes showed a varying degree of stability in our analysis. The MPE, SP-EPN, and PF-SE classes showed a tendency to relapse quite early on. While this did not impair the OS of the affected patients, it does not fit the view of these tumors as rather non-aggressive entities. We were thus able to support similar results of previous analyses focused on these molecular classes [3, 39, 40]. For the EPN-PFA subtypes, the separation between the groups PFA-1 and PFA-2 was possible, but the separation between the smaller molecular subtypes was not as clear, both in the analysis of all samples and when restricted to EPN-PFA tumors only. These results question the reliability of these smaller subtypes when tested in a larger cohort. This might be related to intratumoral heterogeneity and poses the question of whether it might be possible to establish more robust subtypes in the future. However, an even bigger and well-annotated cohort may be necessary. Previous studies have indicated a prognostic value of the subtypes [29]. We showed that they are more accurate when predicting PFS than a prediction based on the broader molecular type. But even inside these more distinguished molecular subtypes, there is still a significant heterogeneity detectable concerning the survival of different patients. Thus, giving an accurate prognosis for a single case still poses a challenge.

However, the classification into types is not the only predictive factor for survival. The localization of the tumor can also be a prognostic factor. Infratentorial EPN-ZFTA tumors have been previously described and we were able to identify 12 cases of EPN-ZFTA located in the posterior fossa in our cohort [8, 17]. They showed an unfavorable prognosis with a 5-year PFS of 25% and a 5-year OS rate of only 56% (Fig. 2j, k). Considering their dismal prognosis and their relative frequency of about 6% in our cohort, these tumors should be included as a differential diagnosis in the group of posterior fossa tumors in infants and children, whereas the loss of *CDKN2A* and *CDKN2B* is a highly predictive marker in supratentorial EPN-ZFTA, it does not seem to contribute to the unfavorable prognosis in the posterior fossa tumors, as all posterior fossa tumors except one had a flat CNV profile for *CDKN2A/B*. In our study, we could not identify whether the impaired prognosis resulted from a molecular or genetic factor inherent to these tumors or due to clinical factors such as difficulties arising during treatment (e.g., inoperability). Another relevant factor could be the early metastasis of these tumors. In our cohort, data on metastases were only available for 3/12 patients with EPN-ZFTA in the posterior fossa. However, all these cases showed no evidence of metastatic disease. This should be subject to further research to improve the outcome of patients with these rare tumors. The identification of these tumors also leads to the conclusion that the restriction of the molecular types to a certain CNS compartment might not be as strict as previously thought. Clinicians and pathologists should thus be open to the possibility that a tumor in a typical localization for one molecular type might express the methylation profile indicative of a molecular type from another localization.

The resection status has generally been regarded as one of the most important factors for the prognosis of patients with ependymoma [4]. We confirmed that an STR indeed leads to an impaired PFS and OS when looking at the whole ependymoma cohort (Suppl. Fig. 3). However, when analyzing the individual molecular types, we found that it might be more important in some subgroups than in others. The EPN-ZFTA type for example did not seem to benefit significantly from a GTR, whereas EPN-PFA showed an improved PFS and OS (Suppl. Fig. 3). For EPN-PFB, the relapse rate was considerably lower in individuals who received a GTR, but the OS was not affected (Suppl. Fig. 3). This shows that the resection status does not necessarily impact the survival of patients with ependymoma, and second surgery might not always provide a benefit to the patient when weighing the risks.

Besides the molecular classification and the localization of the tumor, gains and losses of single genes or whole chromosome arms as indicated by copy number variation profiling have been shown to be relevant to the outcome of patients [2, 12]. In EPN-PFA, both a gain of chromosome 1q and a loss of chromosome 6q are highly predictive of the patient’s survival. However, a simultaneous aberration of these two chromosome arms leads to an even worse prognosis with almost 90% of patients experiencing relapse and only 33% of patients being alive five years after the initial diagnosis (Fig. 3d, e). In EPN-ZFTA, the loss of *CDKN2A* and *CDKN2B* is of high relevance to the survival of patients. Again, this effect is emphasized when both genes are lost. The effects of these changes should be investigated further, as well as their relevance to current therapeutic regimens and potential new treatment strategies.

Machine learning methods based on DNA methylation have already shown promising results in other tumor entities, for example, in identifying the origin of metastases [19, 25]. This makes them a good option to further improve the survival prediction in affected patients. In our work, we showed that a survival prediction simply based on the 10,000 most variable CpG sites already holds a slight advantage over the use of molecular types and subtypes. An integrated regression model combining the methylation scores with clinical factors (age, sex, extent of resection) and copy number alterations further improved these results. More complex models or the addition of further molecular alterations [32] might yield even better results. However, these models are dependent on the availability of the corresponding data, whereas the SVM already achieves high prediction scores on methylation data alone. Adding clinical data only lead to a slight advantage compared to the predictions based only on methylation data.

DNA methylation profiling is becoming widely available and is incorporated into the routine diagnostic workup. The example of the Heidelberg brain tumor classifier shows that machine learning methods can directly impact how the diagnostic workup of tumors changes [6]. They can provide additional information and help interpret the complex data resulting from the extensive analyses now available in research and the diagnostic setting.

Many recent studies on ependymomas have provided a high number of methylation profiles and thus made this retrospective analysis possible. Still, our results are limited by the availability of reliable and extensive annotated data. A high amount of data for training and testing is necessary to further explore the methylation data and their connection to other factors such as age, sex, or the localization of the tumor, and to make more accurate predictions. This is also true for the evaluation of current treatment regimens and new therapy options. The case numbers are difficult to reach for rare tumors, which further emphasizes the importance of national and international collaboration and partnerships.

To conclude, we showed that many of the recent findings on ependymomas are also apparent in a big cohort of 2023 tumors. We highlight the importance of DNA methylation analyses and CNV profiling in these tumors, as well as the potential benefit of employing machine learning methods to identify patterns. We have given an outlook on the potential use of machine learning in the prediction of the prognosis of patients. More refined models may be able to make more accurate predictions in the future and enable clinicians to make more informed treatment decisions.

### Supplementary Information

Below is the link to the electronic supplementary material.

Supplementary file1 (DOCX 10740 kb)

Supplementary file2 (XLSX 220 kb)
