# Supplementary material for: Molecular characteristics and improved survival prediction in a cohort of 2023 ependymomas
Source: Acta Neuropathol. 2024 Jan 24;147(1):24. doi: 10.1007/s00401-023-02674-x (PMC10808151; doi:10.1007/s00401-023-02674-x)
Supplement: Supplementary file 1 — Supplementary file1 (DOCX 10740 kb) [file 401_2023_2674_MOESM1_ESM.docx]

**Supplementary Figures**

Supplementary Figure 1 | Survival across different age groups and molecular types

Supplementary Figure 2 | Survival across different sexes and molecular types

Supplementary Figure 3 | Survival across the resection status and molecular types

Supplementary Figure 4 | Hierarchical clustering of cases with class prediction scores above the threshold of 0.7 and 0.9

Supplementary Figure 5 | Immune infiltration of the molecular types of ependymoma and effect on survival of mean methylation

Supplementary Figure 6 | Extended results of 5-year PFS prediction with a Support Vector Machine

Supplementary Figure 7 | Results of 10-year OS prediction with a Support Vector Machine

Supplementary Figure 1


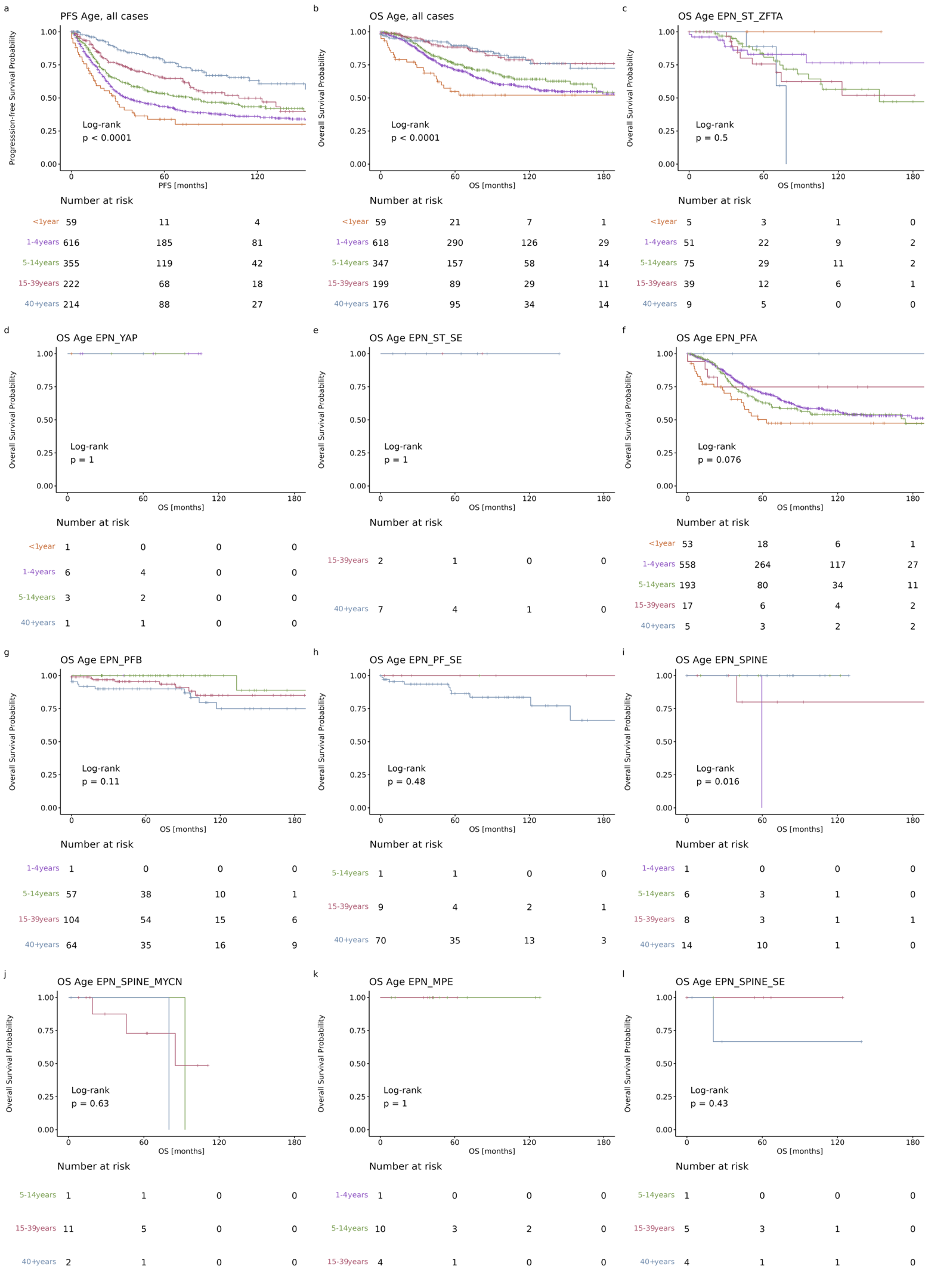


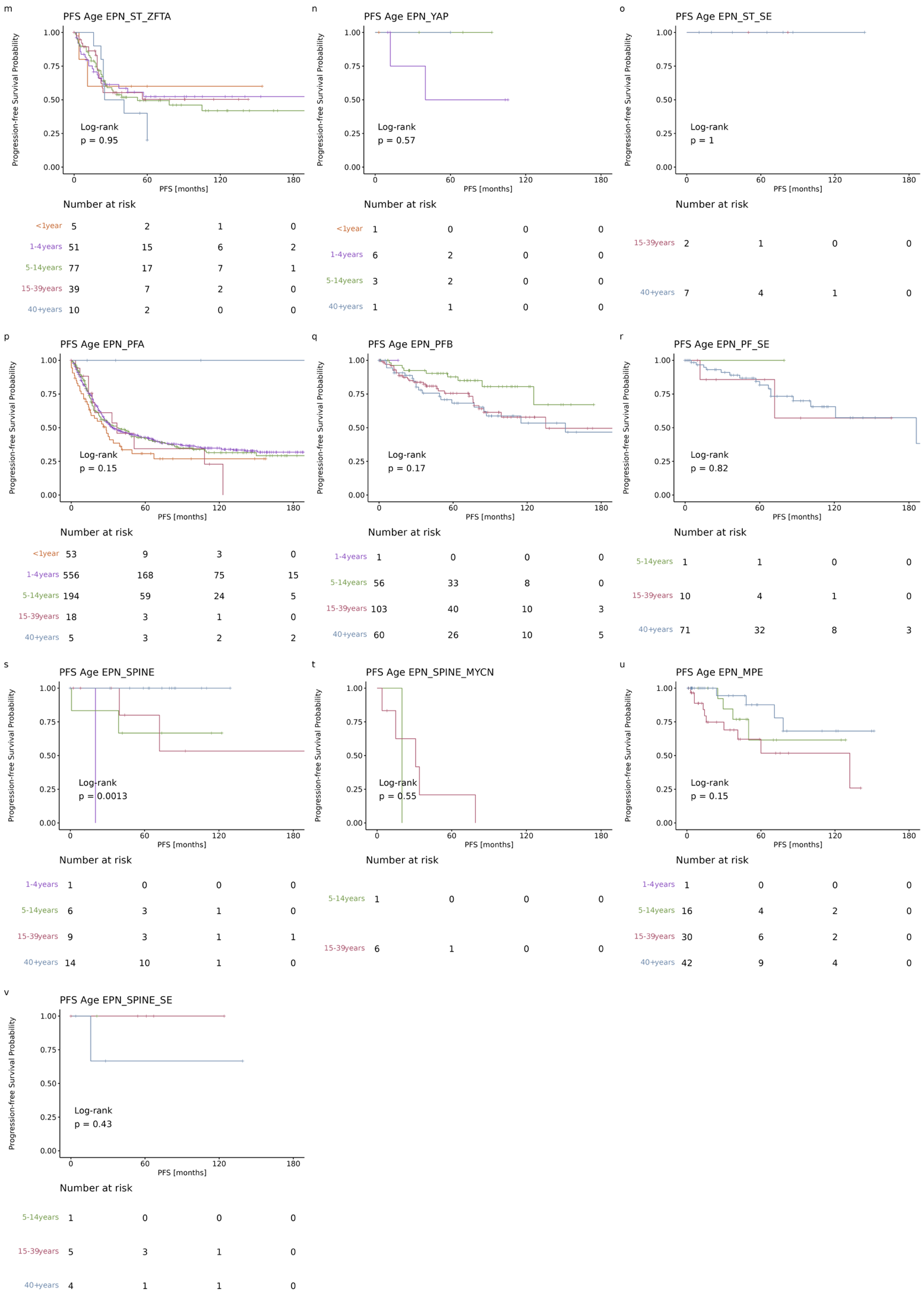


Supplementary Figure 1 | Survival across different age groups and molecular types. (a) Progression-free survival by age group including the full cohort. (b) Overall survival by age group including the full cohort. (c-l) Overall survival of the individual molecular types stratified by age group. (m-v) Progression-free survival of the individual molecular types stratified by age group.

Supplementary Figure 2


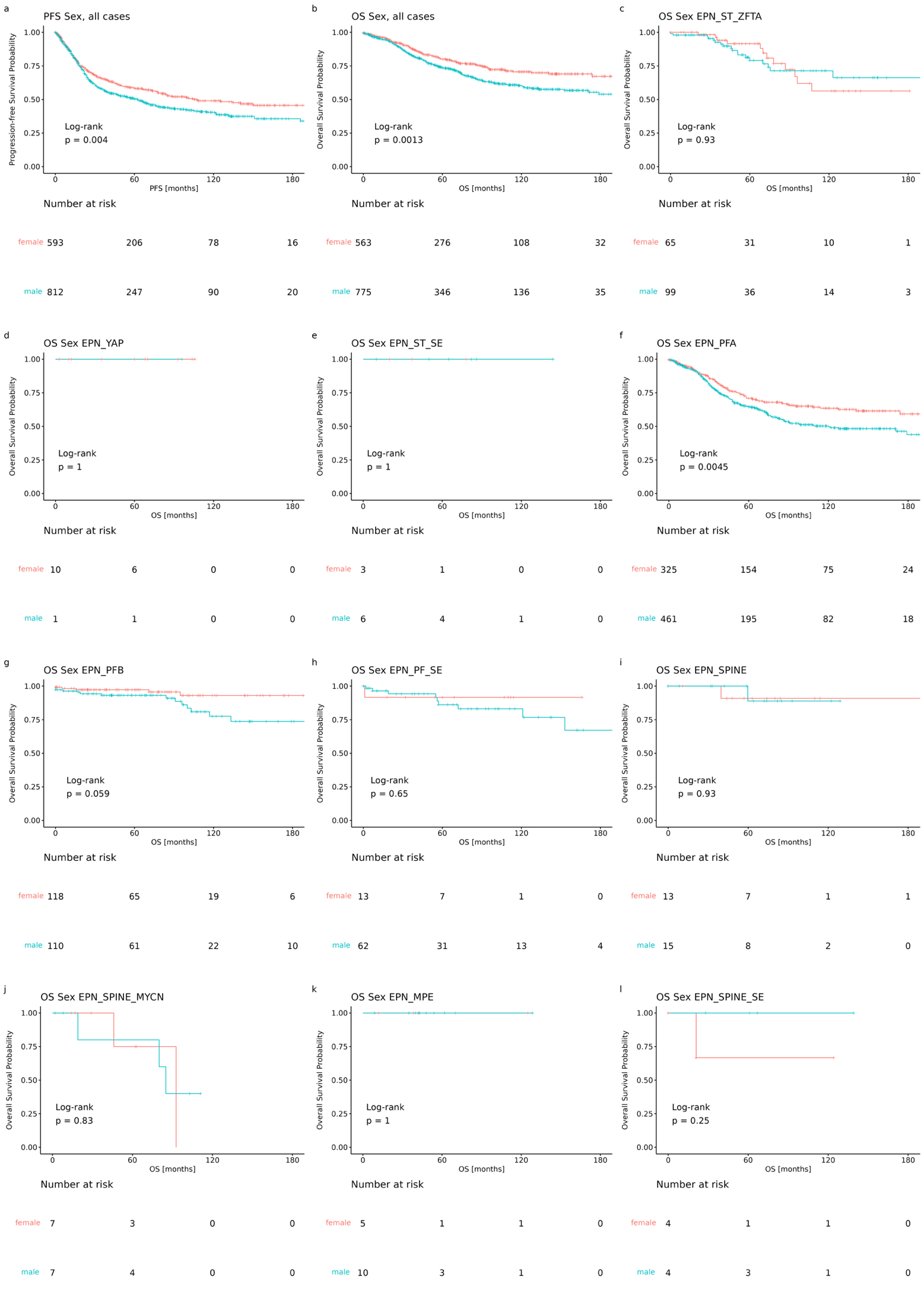


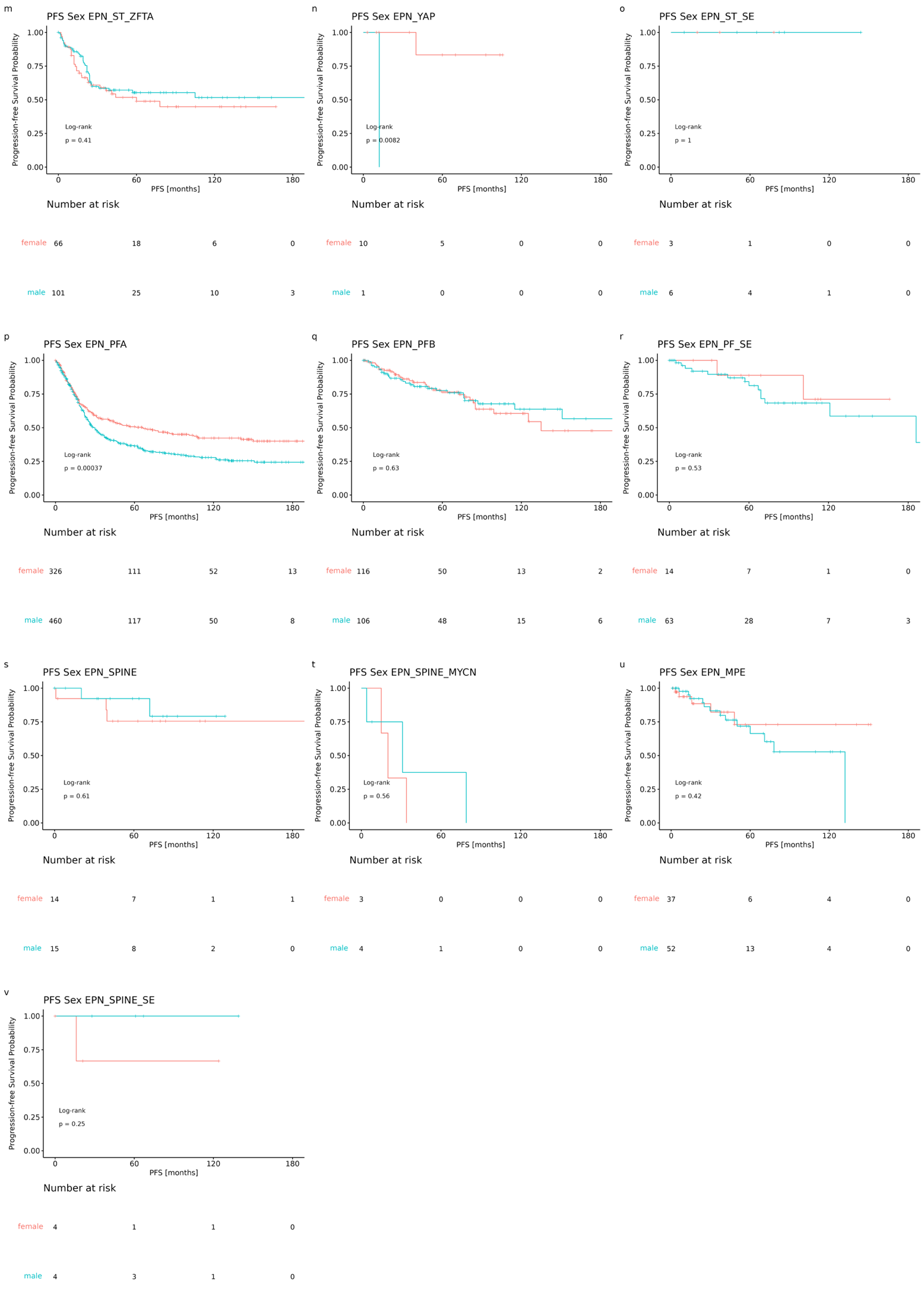


Supplementary Figure 2 | Survival across different sexes and molecular types. (a) Progression-free survival by sex including the full cohort. (b) Overall survival by sex including the full cohort. (c-l) Overall survival of the individual molecular types stratified by sex. (m-v) Progression-free survival of the individual molecular types stratified by sex.

Supplementary Figure 3


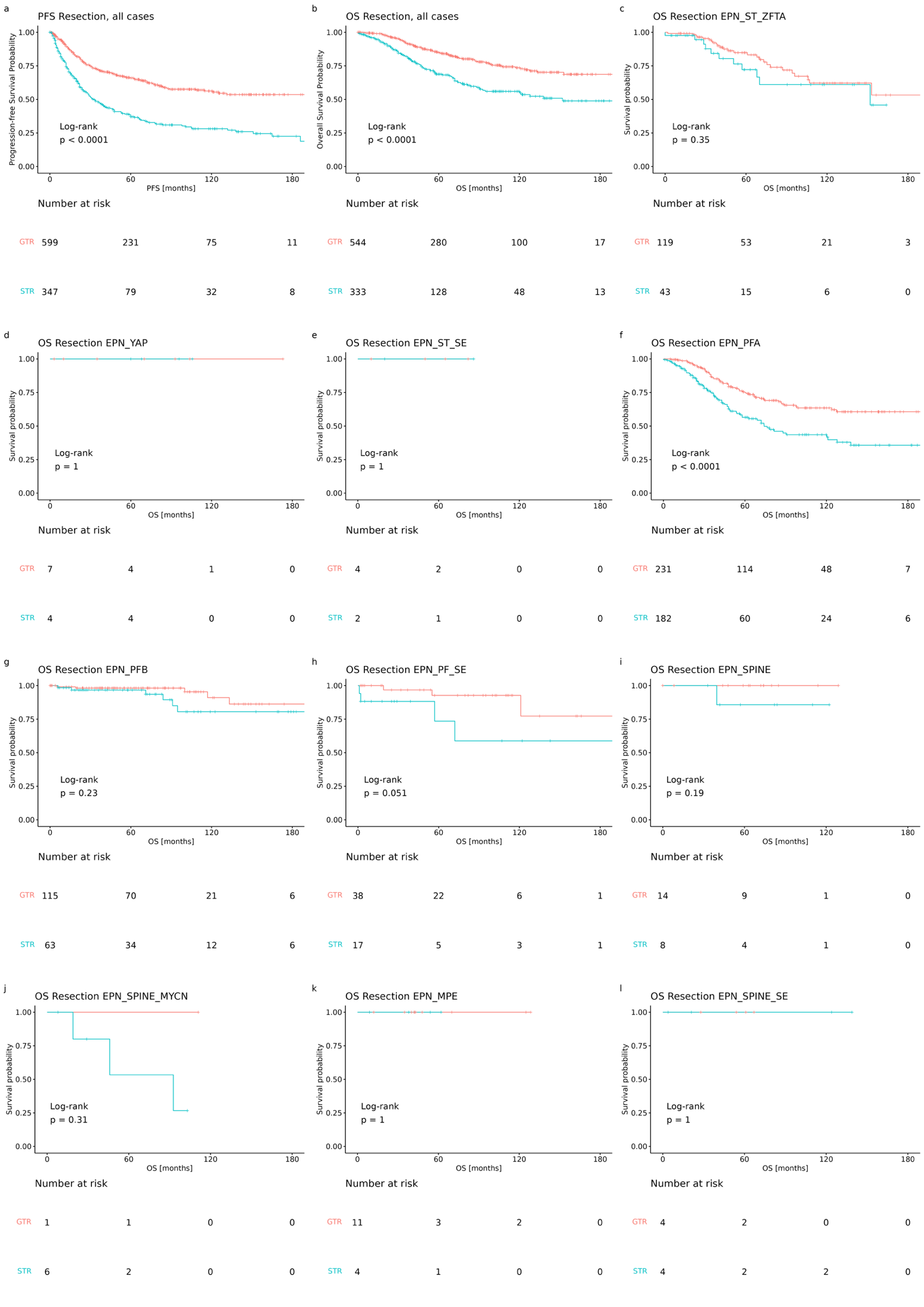


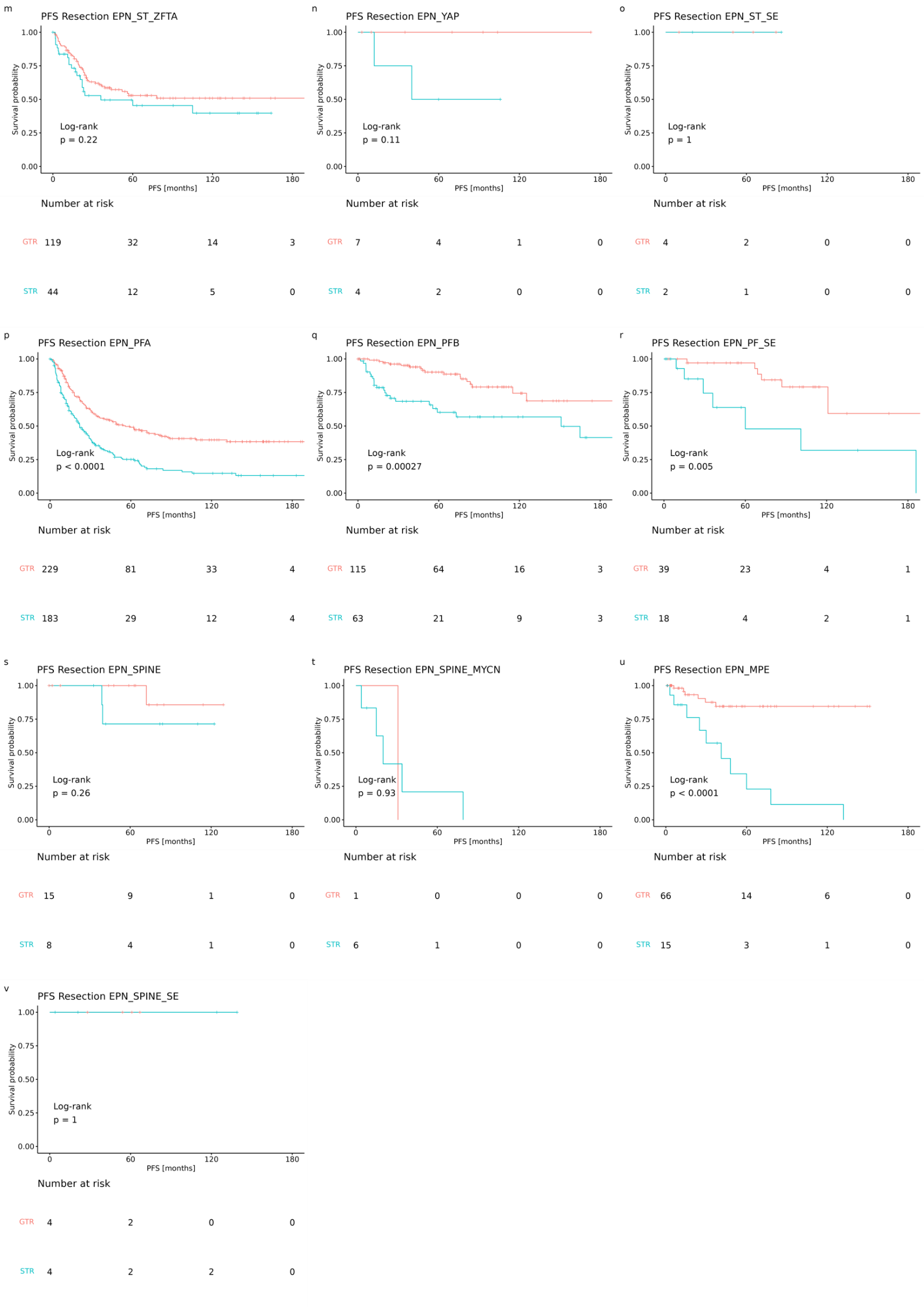


Supplementary Figure 3 | Survival across the resection status and molecular types. (a) Progression-free survival by resection status including the full cohort. (b) Overall survival by resection status including the full cohort. (c-l) Overall survival of the individual molecular types stratified by resection status. (m-v) Progression-free survival of the individual molecular types stratified by resection status.

Supplementary Figure 4


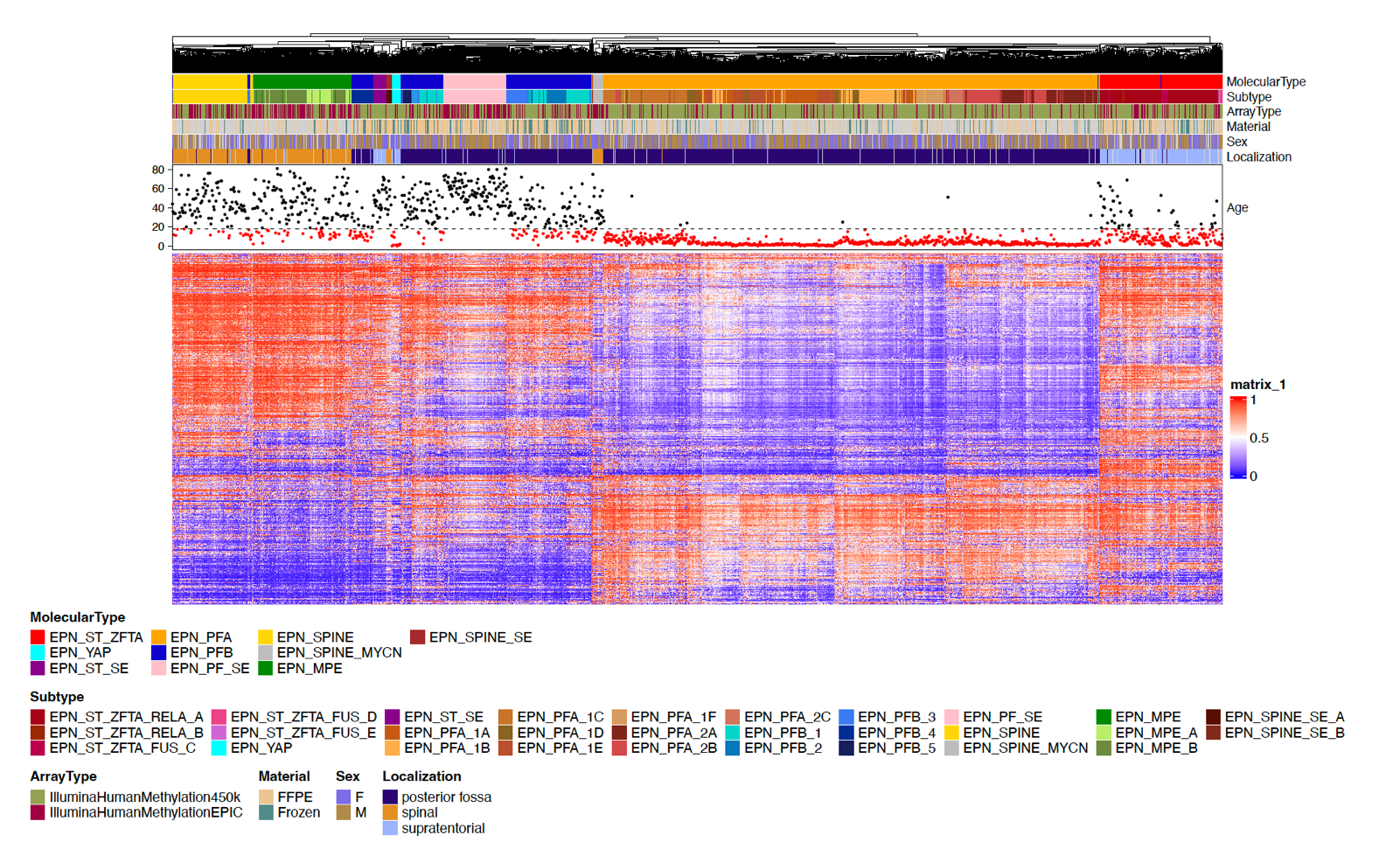


a


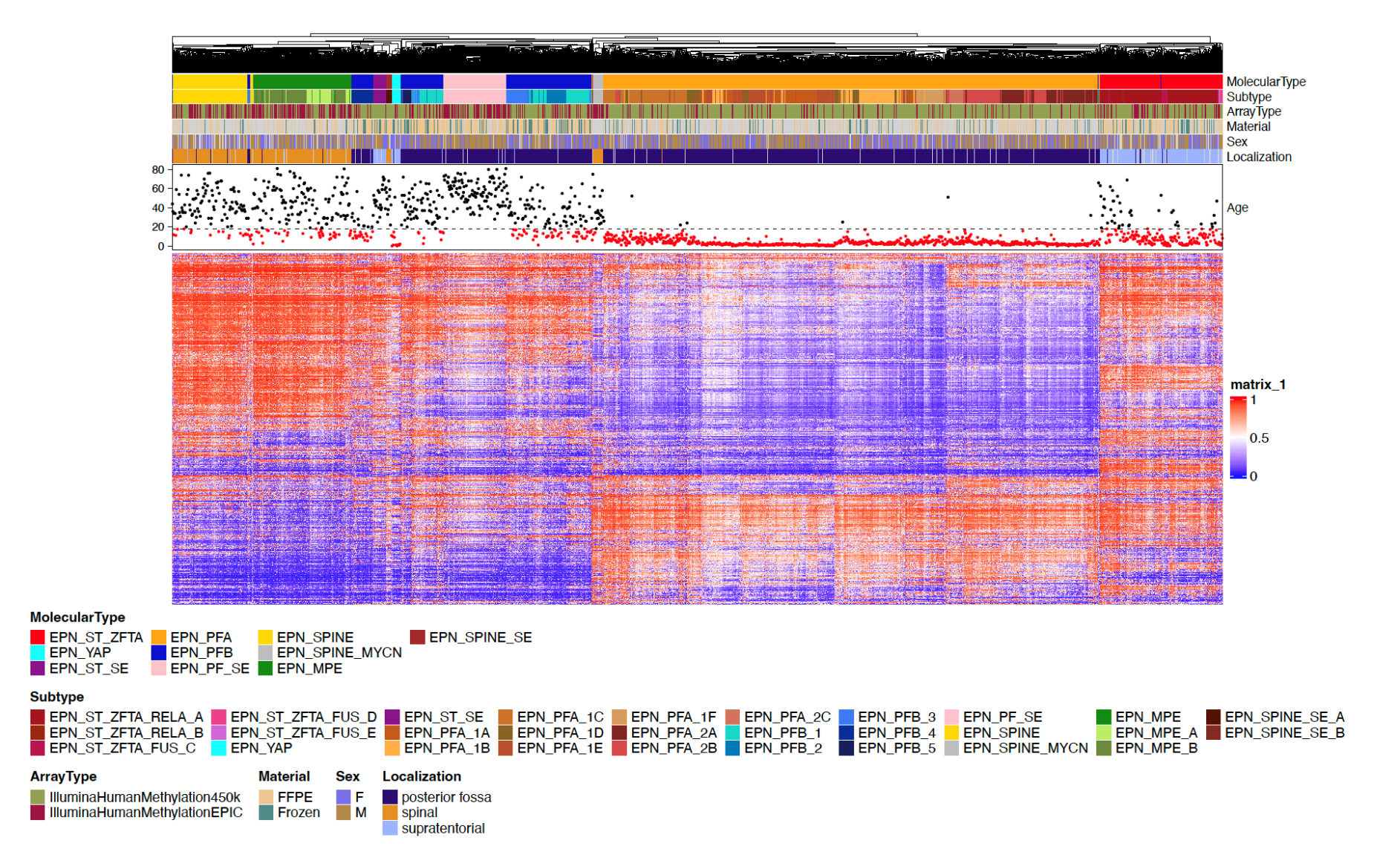


b


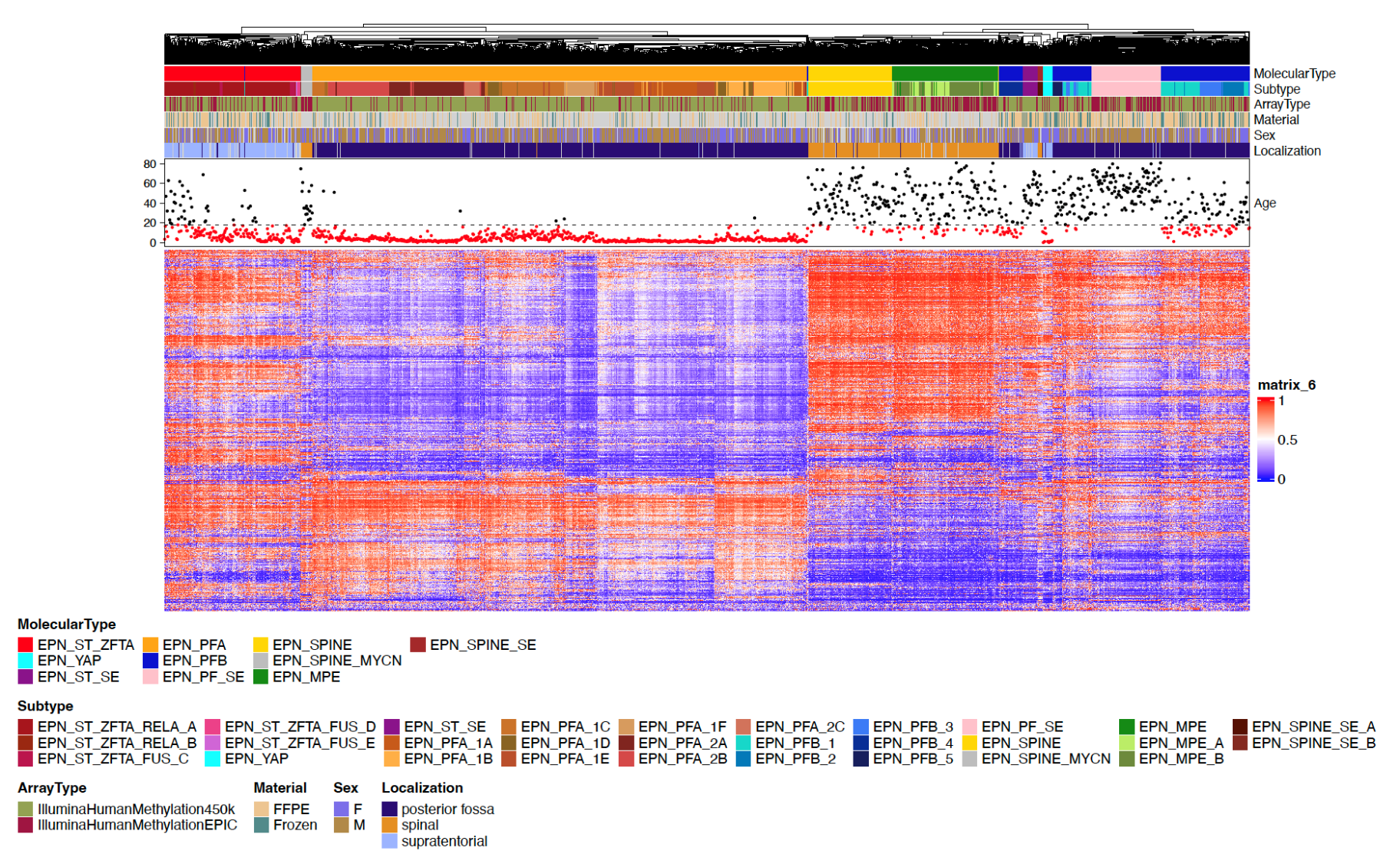


Supplementary Figure 4 | Hierarchical clustering of cases with class prediction scores above the threshold of 0.7 and 0.9. (a) Heatmap showing the hierarchical clustering of DNA methylation profiles of the cases with a classification score above 0.7 in the random-forest based classification (n = 1,884). (b) Heatmap showing the hierarchical clustering of DNA methylation profiles of the cases with a classification score above 0.9 in the random-forest based classification (n = 1,722).

Supplementary Figure 5

a


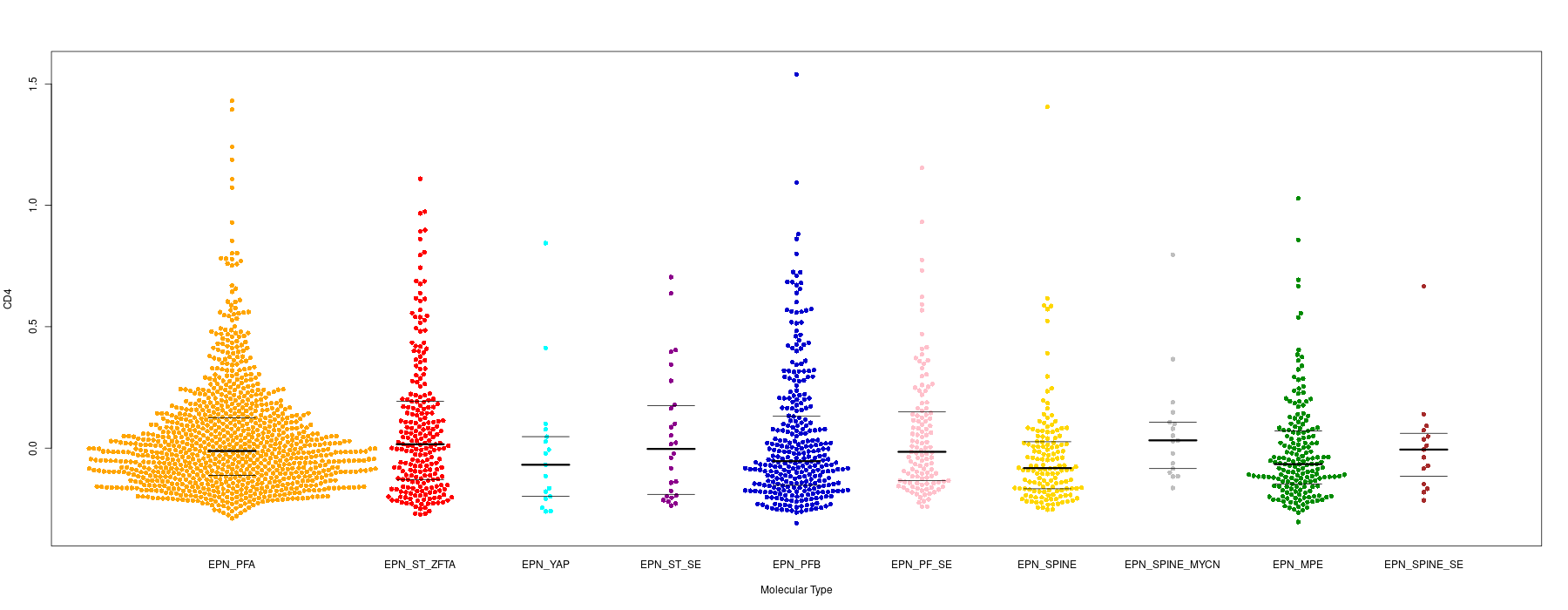


b


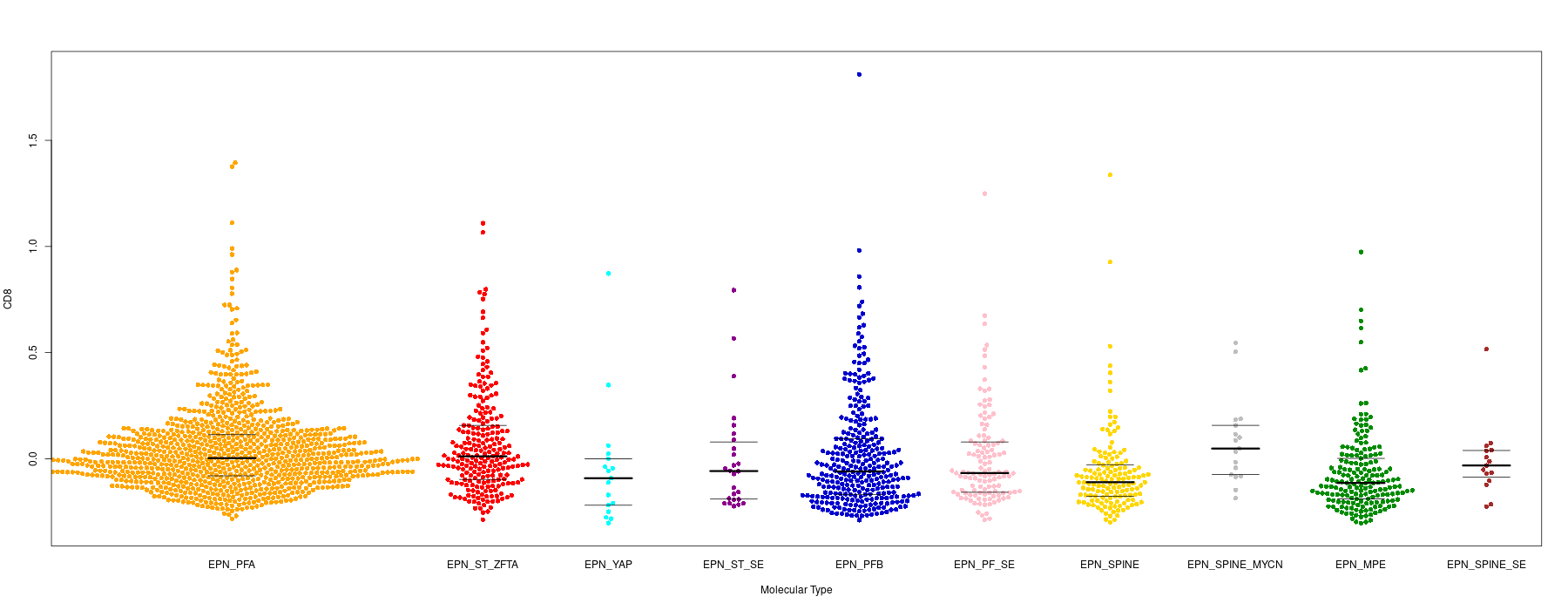


c


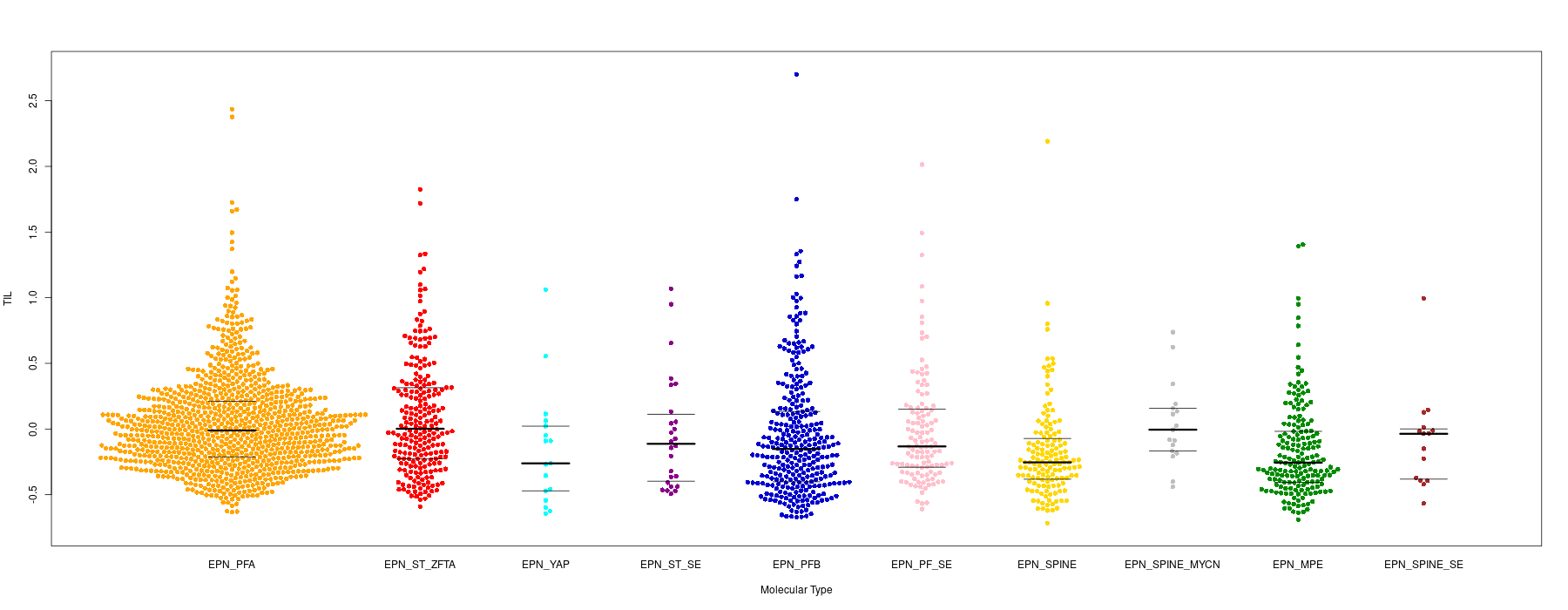


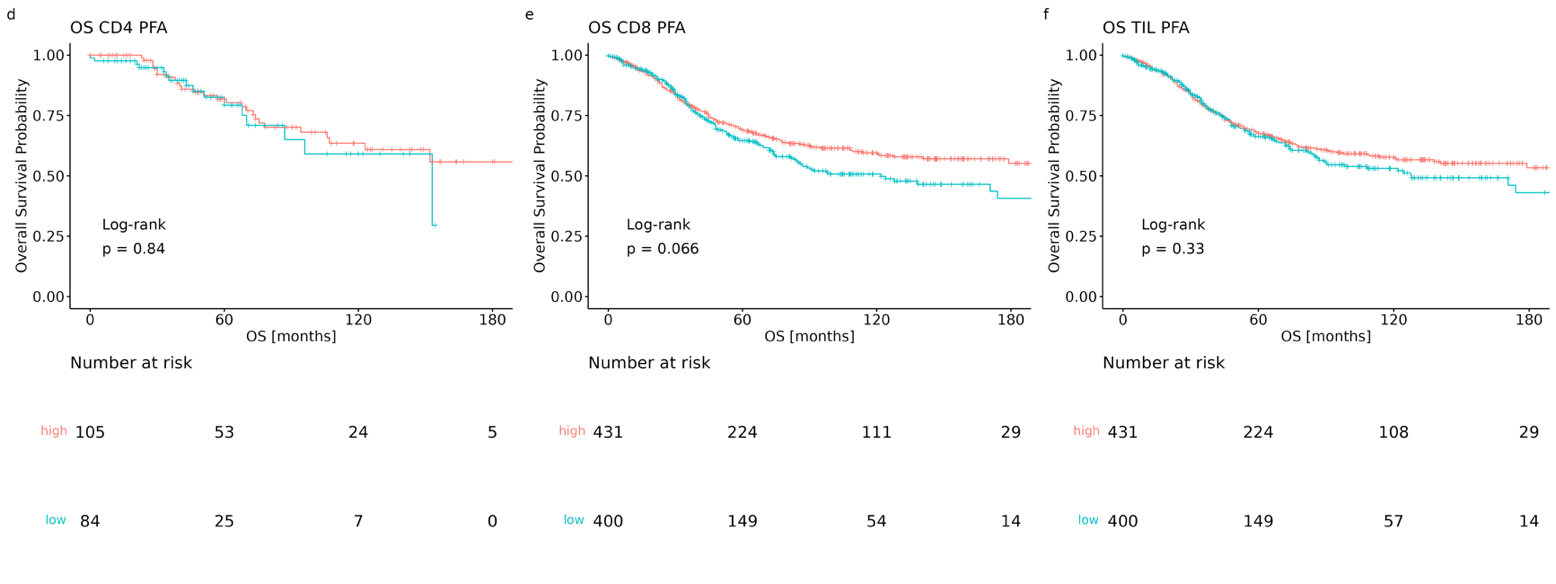


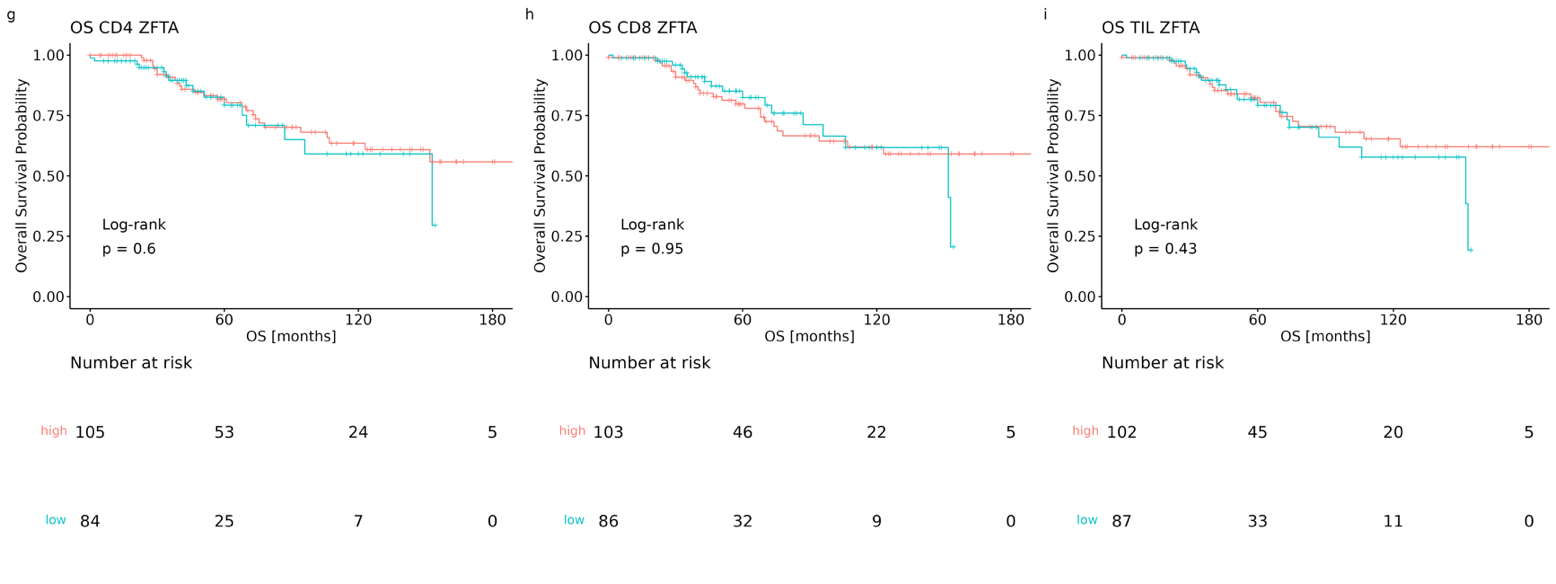


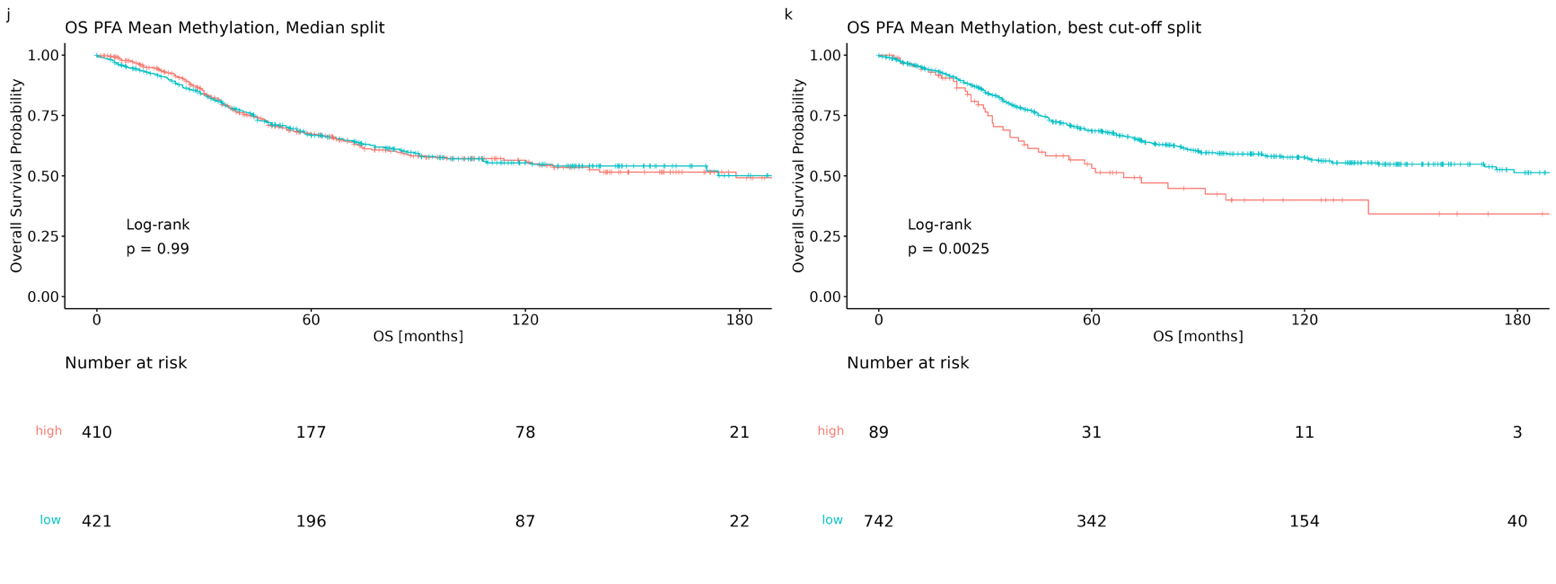


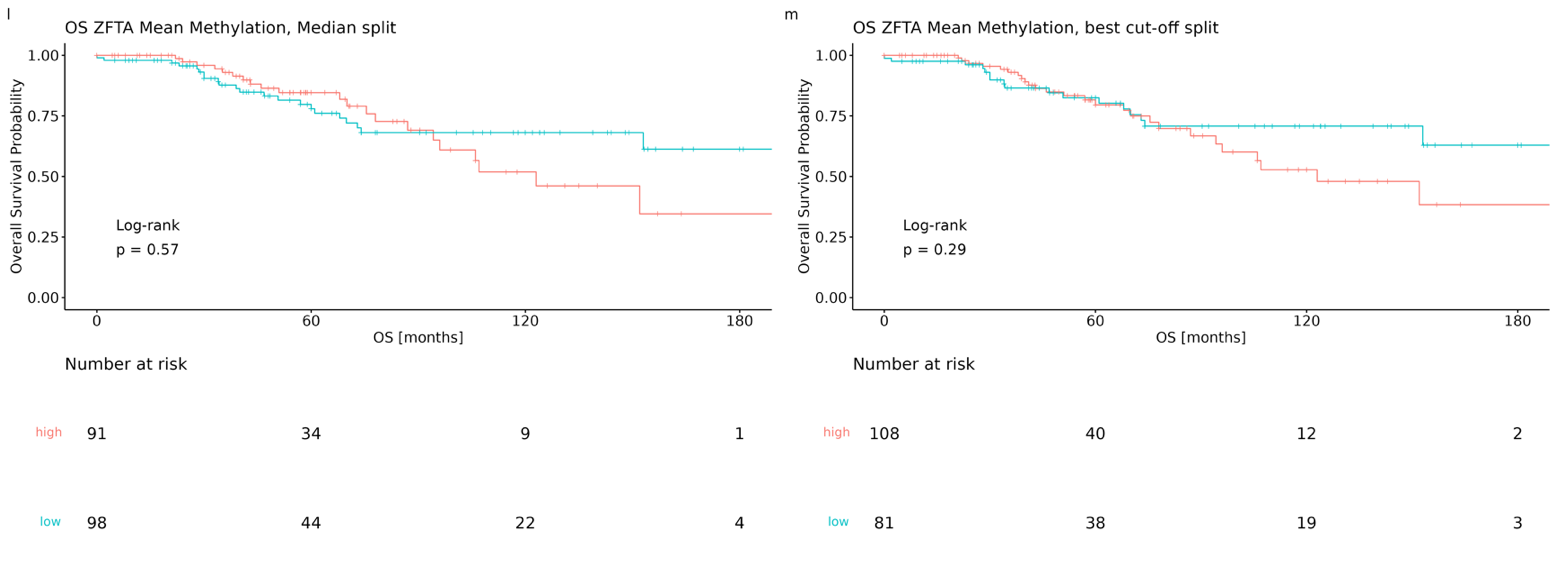


Supplementary Figure 5 | Immune infiltration of the molecular types of ependymoma and effect on survival of mean methylation. (a-c) Beeswarm plots of the immune infiltration with (a) CD4+ cells, (b) CD8+ cells, and (c) tumor-infiltrating lymphocytes (TIL) for the molecular types. (d-f) Survival of EPN-PFA patients with high and low amounts of immune infiltration of (d) CD4+ cells, (e) CD8+ cells, and (f) TILs, split at the median. (g-i) Survival of EPN-ZFTA patients with high and low amounts of immune infiltration of (g) CD4+ cells, (h) CD8+ cells, and (i) TILs, split at the median. (j) Overall survival of EPN-PFA patients with high and low mean methylation split at the median. (k) Overall survival of EPN-PFA patients with high and low mean methylation split at the best statistical cut-off. (l) Overall survival of EPN-ZFTA patients with high and low mean methylation split at the median. (m) Overall survival of EPN-ZFTA patients with high and low mean methylation split at the best statistical cut-off.

Supplementary Figure 6


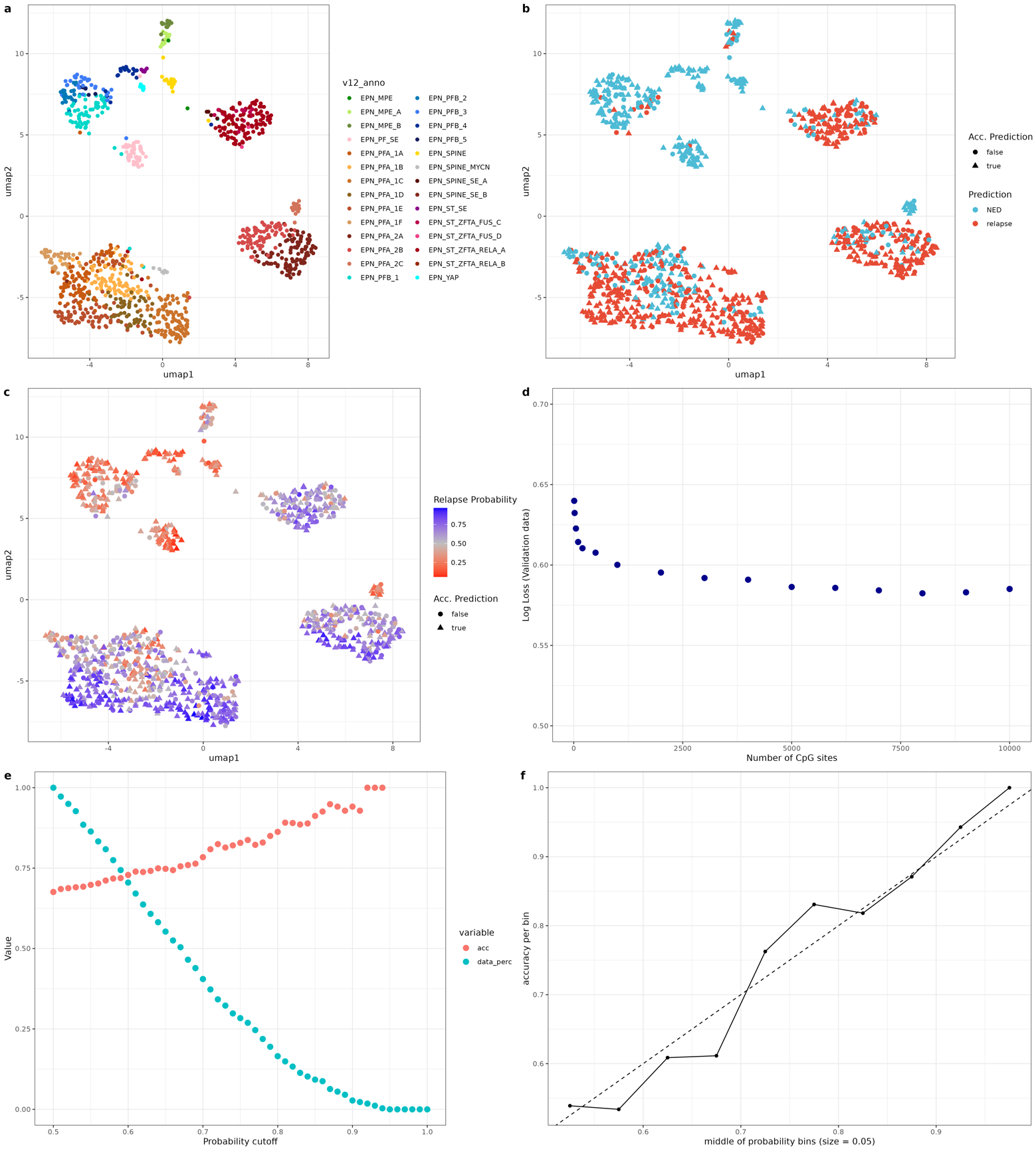


Supplementary Figure 6 | Extended results of 5-year PFS prediction with a Support Vector Machine. (a) UMAP of cases available for 5-year PFS prediction colored according to subtype. (b) UMAP of all predicted cases, the accuracy of prediction indicated by shape: circle = false prediction, triangle = true prediction, color indicating prediction result: blue = NED, red = relapse. (c) UMAP of all predicted cases, color indicating the relapse probability assigned by the SVM, and shape indicating the accuracy of the prediction. (d) Plot showing the Log Loss per number of CpG sites included in the prediction. (e) Accuracy of prediction and percentage of data left, when including cases with a probability above a cutoff between 0.5 and 1.0. (f) Calibration Plot showing the accuracy per probability bin.

Supplementary Figure 7


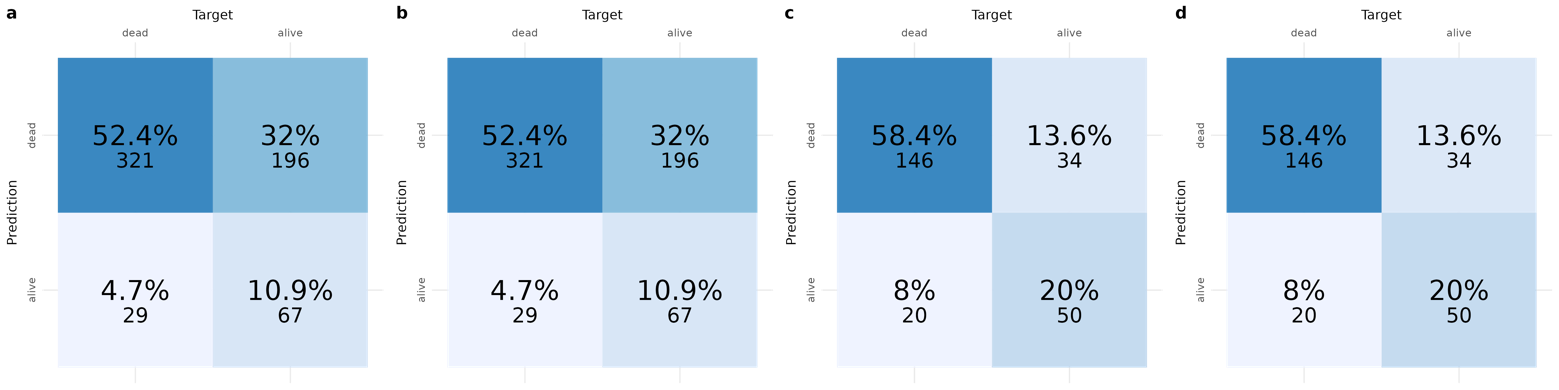


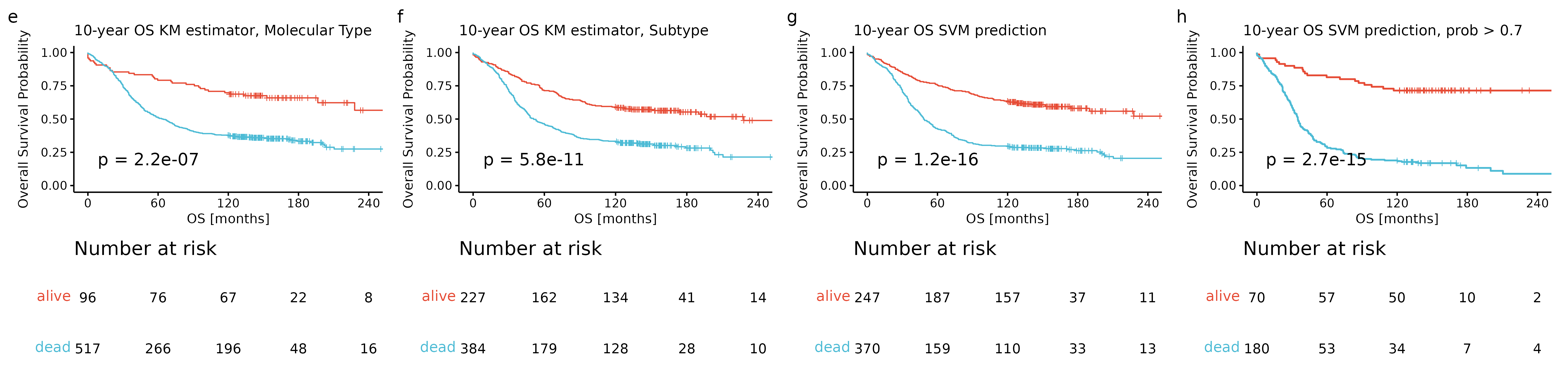


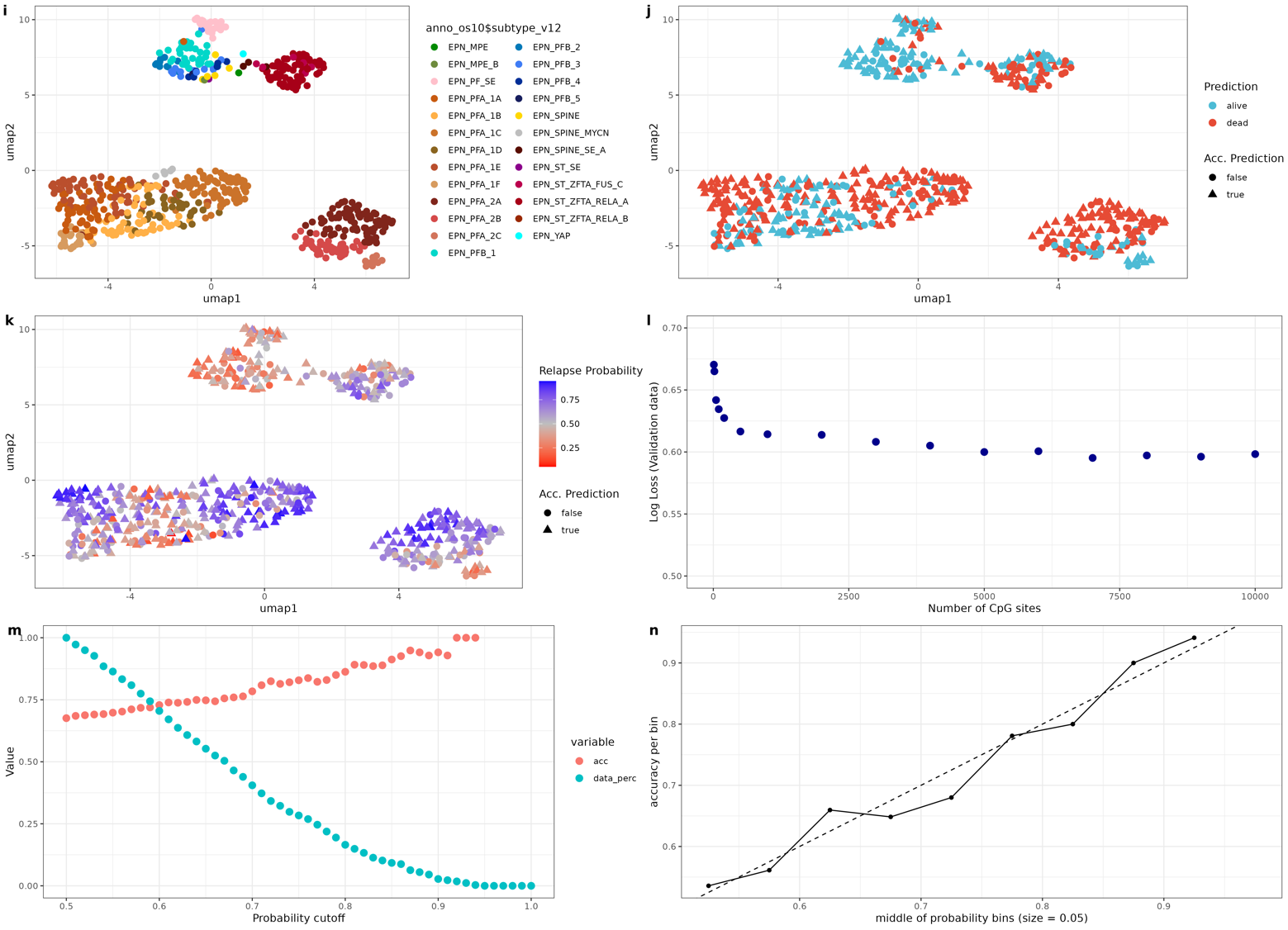


Supplementary Figure 7 | Results of 10-year OS prediction with a Support Vector Machine (SVM). (a-d) Overall survival stratified by the predicted 10-year OS based on: (a) the molecular type, (b) the molecular subtype, (c) the SVM, (d) the SVM predictions with a probability score below 0.3 or above 0.7. (e-h) Confusion matrices and balanced accuracy for the predicted 10-year OS based on: (e) the molecular type, (f) the molecular subtype, (g) the SVM, and (h) the SVM predictions with a probability score below 0.3 or above 0.7. (i) UMAP of cases available for 10-year OS prediction colored according to subtype. (j) UMAP of all predicted cases, the accuracy of prediction indicated by shape: circle = false prediction, triangle = true prediction, color indicating prediction result: blue = alive, red = dead. (k) UMAP of all predicted cases, color indicating the relapse probability assigned by the SVM, and shape indicating the accuracy of the prediction. (l) Plot showing the Log Loss per number of CpG sites included in the prediction. (m) Accuracy of prediction and percentage of data left, when including cases with a probability above a cutoff between 0.5 and 1.0. (n) Calibration Plot showing the accuracy per probability bin.
